# PABPN1 aggregation is driven by Ala expansion and poly(A)-RNA binding, leading to CFIm25 sequestration that impairs alternative polyadenylation

**DOI:** 10.1016/j.jbc.2023.105019

**Published:** 2023-07-07

**Authors:** Wen-Liang Guan, Lei-Lei Jiang, Xiao-Fang Yin, Hong-Yu Hu

**Affiliations:** 1 State Key Laboratory of Molecular Biology, Shanghai Institute of Biochemistry and Cell Biology, Center for Excellence in Molecular Cell Science, Chinese Academy of Sciences, Shanghai, China; 2 University of Chinese Academy of Sciences, Beijing, China

**Keywords:** PABPN1, alanine expansion, poly(A) RNA, nuclear speckles, aggregates, sequestration, alternative polyadenylation

## Abstract

Poly(A)-binding protein nuclear 1 (PABPN1) is an RNA-binding protein localized in nuclear speckles, while its alanine (Ala)-expanded variants accumulate as intranuclear aggregates in oculopharyngeal muscular dystrophy. The factors that drive PABPN1 aggregation and its cellular consequences remain largely unknown. Here, we investigated the roles of Ala stretch and poly(A) RNA in the phase transition of PABPN1 using biochemical and molecular cell biology methods. We have revealed that the Ala stretch controls its mobility in nuclear speckles, and Ala expansion leads to aggregation from the dynamic speckles. Poly(A) nucleotide is essential to the early-stage condensation that thereby facilitates speckle formation and transition to solid-like aggregates. Moreover, the PABPN1 aggregates can sequester CFIm25, a component of the pre-mRNA 3′-UTR processing complex, in an mRNA-dependent manner and consequently impair the function of CFIm25 in alternative polyadenylation. In conclusion, our study elucidates a molecular mechanism underlying PABPN1 aggregation and sequestration, which will be beneficial for understanding PABPN1 proteinopathy.

Poly(A)-binding proteins are a major class of RNA-binding proteins (RBPs) that mediate the functions of poly(A) tails of mRNAs (1, 2). Among them, poly(A)-binding protein nuclear 1 (PABPN1) is a ubiquitously expressed RBP involved in polyadenylation of pre-mRNAs (3, 4, 5). It is mostly concentrated in nuclear speckles, which are ribonucleoprotein condensates consisting of splicing factors, poly(A) mRNAs, and other component RBPs (6, 7, 8, 9). The WT PABPN1 protein possesses a stretch of ten alanine (10Ala, 10A) residues in its N-terminus, whereas the alanine (Ala) stretch may have an expansion to 11 ∼ 18 residues in the pathogenic forms (10, 11, 12). Ala-expanded PABPN1 is able to form protein aggregates in nucleus, which may cause an autosomal-dominant disease, oculopharyngeal muscular dystrophy (OPMD) (11, 12). However, the mechanism responsible for PABPN1 aggregation and disease pathology remains largely unknown.

In eukaryotes, the mRNA 3′-end processing is important in the regulation of gene expression (13). The core pre-mRNA 3′-end processing complex consists of four subcomplexes, including cleavage and polyadenylation specificity factor, cleavage stimulation factor, cleavage factor I and cleavage factor II, as well as some other proteins (14, 15). The processing occurs in two steps: firstly, alternative endonucleolytic cleavage that cleaves the pre-mRNA at the poly(A) site by cleavage factors and secondly, pre-mRNA polyadenylation that synthesizes a poly(A) tail by poly(A) polymerase (PAP) (15, 16). Early studies showed that PABPN1 as a stimulator is essential for nascent mRNA polyadenylation and control of poly(A) tail length, in which PABPN1 binds nascent poly(A) tails and increases PAP activity by stabilizing the interaction of PAP with RNA (5, 17). Emerging evidence has suggested that PABPN1 is also involved in suppressing alternative polyadenylation (APA) (4) in which it directly binds proximal polyadenylation sites (PASs) and competes with the cleavage factors, while knock-down of PABPN1 may result in more proximal PAS used and 3′-UTR shortening. Similar result has also been obtained in a mouse model expressing the OPMD-related 17A-expanded variant (18). However, how Ala expansion of PABPN1 influences the APA function remains elusive. An early study reported that PABPN1 directly interacts with CFIm25, a 25-kDa component of the mammalian cleavage factor I complex, which has a preference to use the distal PAS (19).

Mislocalization and aggregate formation of mutant RBPs, such as TDP-43, FUS, and hnRNP A1, are commonly pathological hallmarks in various neurodegenerative diseases (20). Aggregation of these RBPs is thought to be closely related to aberrant phase separation (PS) (21, 22). Among, SRSF2 (SC35), a key component of nuclear speckles (23), has also been found to form droplet-like condensates *via* PS (24). PABPN1 contains an RNA-recognition motif (RRM) that has a high affinity to poly(A) tails of mRNAs (10). Previous studies revealed that localization of PABPN1 (herein called PABP2) in nuclear speckles depends on binding to poly(A) mRNAs (7, 8). Indeed, RNA plays a critically important role in PS or condensation of some RBPs (22, 25). However, it is still largely unknown whether PABPN1 undergoes PS in the formation of nuclear speckles and how poly(A) mRNAs regulate its assembling into the nuclear speckles.

Unlike most mutant RBPs that form cytoplasmic aggregates or inclusions (26, 27, 28), the Ala-expanded variants of PABPN1 do not undergo mislocalization to cytoplasm but accumulate in the nucleus. Since the morphologies and molecular features of WT PABPN1 and its Ala-expanded variants were not much different in the cultured cells, some studies regarded the nuclear puncta formed by the 17A variant as solid-like aggregates (29). So, distinguishing these puncta becomes to a prerequisite for elucidating the mechanism underlying the pathogenic Ala expansion. Even so, Ala-expanded PABPN1 can form KCl-insoluble nuclear aggregates in mouse models, due to the aggregates resistant to salt extraction (30, 31, 32). These studies concentrate on the cellular effects of PABPN1 aggregates but few on characterizing the features of the speckles or aggregates formed by the PABPN1 variants at molecular level. In addition, although it is widely accepted that Ala expansion leads to aggregation in mouse models or OPMD patients, evidence from different groups has suggested that PABPN1 aggregation is independent on the Ala stretch (33, 34, 35). Even there are some reports which shown that a complete deletion of the polyalanine tract gives no effect on the formation of aggregates in their cultured cells (33, 34). As known, the distinct pathological hallmark of OPMD is the presence of fibrillar intranuclear inclusions of PABPN1 in the skeletal muscle cells of patients (11), but the cellular effects due to abnormal accumulation of PABPN1 aggregates remain unexplored.

In this study, we sought to address two important questions: what factors drive PABPN1 phase transition from nuclear speckles to aggregates and what is the cellular consequence of PABPN1 aggregation? To this end, we combined the studies in cell model and *in vitro* to elucidate the roles of Ala stretch and poly(A) mRNA in phase transition of PABPN1 and their effects on APA. Our results demonstrate that Ala expansion triggers PABPN1 to undergo phase transition from dynamic nuclear speckles to solid-like aggregates and poly(A) mRNA may modulate the phase behaviors. We have also revealed that the Ala-expanded PABPN1 aggregates result in alteration of the PAS usage through sequestering CFIm25 and consequently disrupting its function in APA. This study provides potential mechanisms underlying phase transition of PABPN1, sequestration of cellular essential factors, and deleterious alteration of pre-mRNA 3′-UTR processing, which may be beneficial to understanding of the cytotoxicity and proteinopathy of PABPN1.

## Results

### Ala expansion enhances aggregate formation of PABPN1 in nucleus

**Figure 1:**
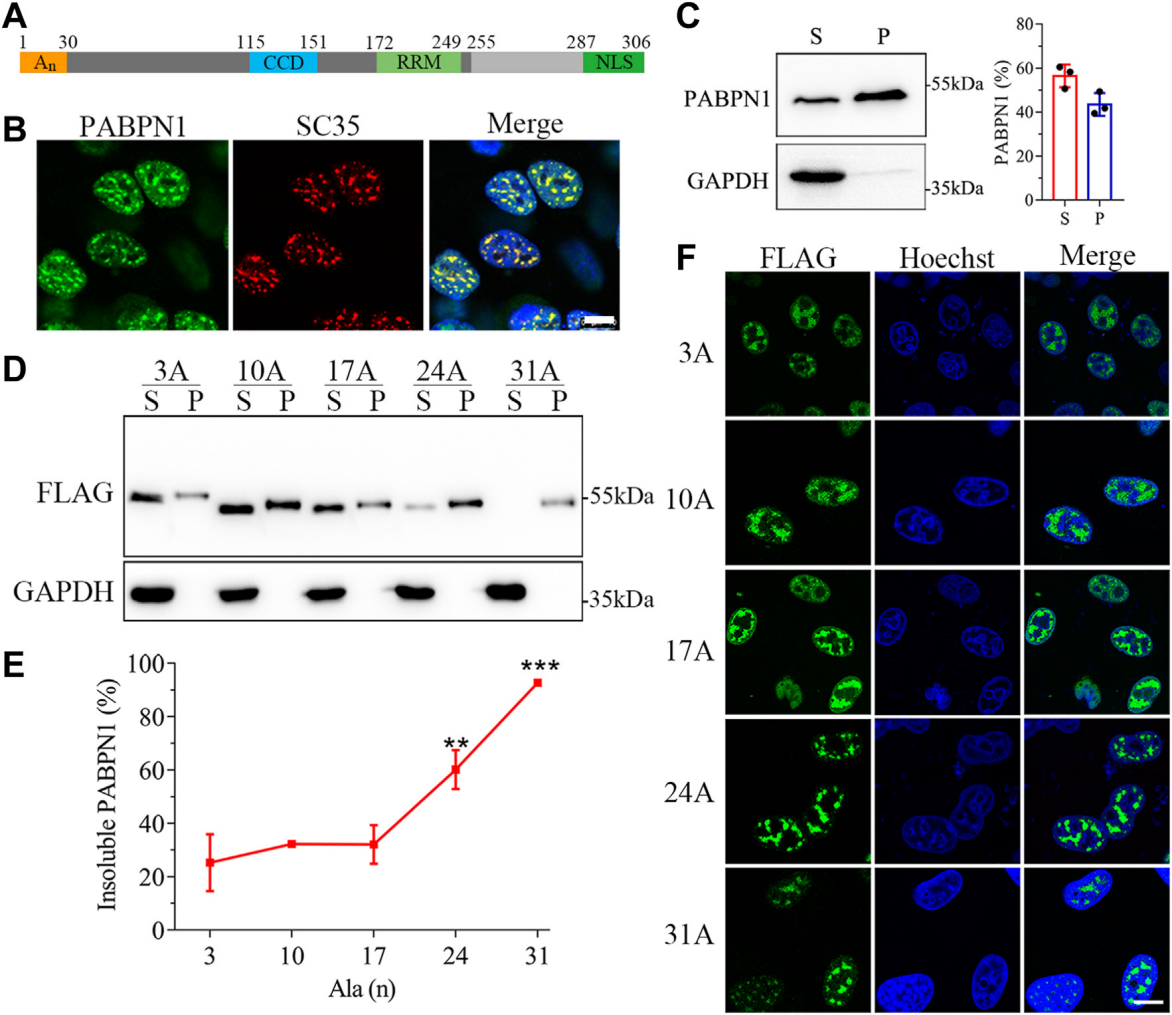
**Ala expansion alters the phase behaviors of PABPN1.***A*, domain architectures of PABPN1. *B*, immunofluorescence imaging of endogenous PABPN1 colocalized with SC35 in HeLa cells. Cells were fixed and immunostained with anti-PABPN1 and anti-SC35 antibodies after culture for 48 h. Endogenous PABPN1, *green*; SC35, *red*; Hoechst, *blue*. Scale bar represents 10 μm. *C*, S/P fractionation for endogenous PABPN1. HeLa cells were cultured for 48 h, and then the cell lysates were subjected to S/P fractionation and Western blotting. Since the volume of the supernatant was 2-fold larger than that of the pellet, the supernatant and pellet fractions of PABPN1 (%) were estimated by the formulas: F_S_ (%) = 2S/(2S + P)∗100%, F_P_ (%) = P/(2S + P)∗100%. Data are presented as mean ± SD (n = 3). *D*, S/P fractionation for Ala-expanded variants of PABPN1. The FLAG-tagged PABPN1 variants were transiently expressed in HeLa cells, and 48-h later, S/P fractionation followed by Western blotting were performed. *E*, quantitative analysis of the insoluble fraction in (*D*). F_P_ (%) = P/(2S + P)∗100%. Data are presented as mean ± SD (n = 3). ∗∗, *p* < 0.01, 24A *versus* 10A; ∗∗∗*p* < 0.001, 31A *versus* 10A. *F*, immunofluorescence imaging of the Ala-expanded PABPN1 variants in HeLa cells. Cells were fixed and immunostained with an anti-FLAG antibody 48 h after transfection. FLAG, *green*; Hoechst, *blue*. Scale bar represents 10 μm. A_n_, Ala stretch; CCD, coiled-coil domain; RRM, RNA-recognition motif; NLS, nuclear localization signal; PABPN1, Poly(A)-binding protein nuclear 1; S/P, supernatant/pellet.

The PABPN1 protein possesses an RRM domain, a coiled-coil domain (CCD), a nuclear localization signal (NLS) at its C-terminus, and more importantly an Ala stretch at the N-terminus (Fig. 1*A*). The intranuclear aggregates or inclusions formed by Ala-expanded PABPN1 are commonly believed to contribute to the pathogenesis of OPMD disease (11, 12). Given that normal PABPN1 is located in the nuclear speckles that are generally thought to undergo PS (36, 37), we firstly analyzed subcellular localization of endogenous PABPN1 in HeLa cells by immunofluorescence imaging. Consistent with the previous observation (7, 8), PABPN1 formed roughly rounded puncta in nucleus and was completely colocalized with SC35, a nuclear speckle marker (Fig. 1*B*). Next, we performed supernatant/pellet (S/P) fractionation experiment to examine whether the PABPN1 speckles were soluble or insoluble. It showed that endogenous PABPN1 was partitioned in both soluble supernatant (∼57%) and insoluble pellet (∼43%) (Fig. 1*C*), compared to GAPDH that only existed in the supernatant fraction. It implies that the PABPN1 speckles are rather sedimentable so as to form partly insoluble accumulates in nucleus.

To study the effect of Ala expansion on its phase transition, we prepared a series of constructs with different Ala-residue length at the N-terminus, namely PABPN1-3A, 10A (WT), 17A, 24A, and 31A. We firstly checked the total protein levels of these PABPN1 variants in HeLa cells, demonstrating that all the PABPN1 variants were expressed at similar levels (Fig. S1). S/P fractionation showed that, as WT (10A) (c.a. 32%), the 3A and 17A variants were partially enriched into the pellet fractions, whereas 24A and 31A had their insoluble proportions increased significantly (Fig. 1, *D* and *E*). Especially, the soluble fraction was sparsely detected in the 31A variant, suggesting that 31A had assembled into insoluble aggregates almost completely (Fig. 1*D*). Notably, the total protein level of 31A was much lower than those of the others, probably due to cytotoxicity of the aggregates. It seemed that the aggregation ability is roughly proportional to the length of Ala stretch in PABPN1 when the number of Ala residues is over 17 (Fig. 1*E*). To our surprise, the aggregation ability of the 17A variant showed almost no significant difference to that of 10A (WT) or 3A, although it was reported to be pathogenic in muscles (10, 11). Immunofluorescence imaging also confirmed the above observation that all the Ala variants could form small puncta in HeLa cells (Fig. 1*F*). These results suggest that Ala expansion indeed enhances aggregate formation of PABPN1 in nucleus, and generally, longer Ala stretch may render the protein stronger aggregation propensities.

### Ala expansion reduces the mobilities of PABPN1 puncta

**Figure 2:**
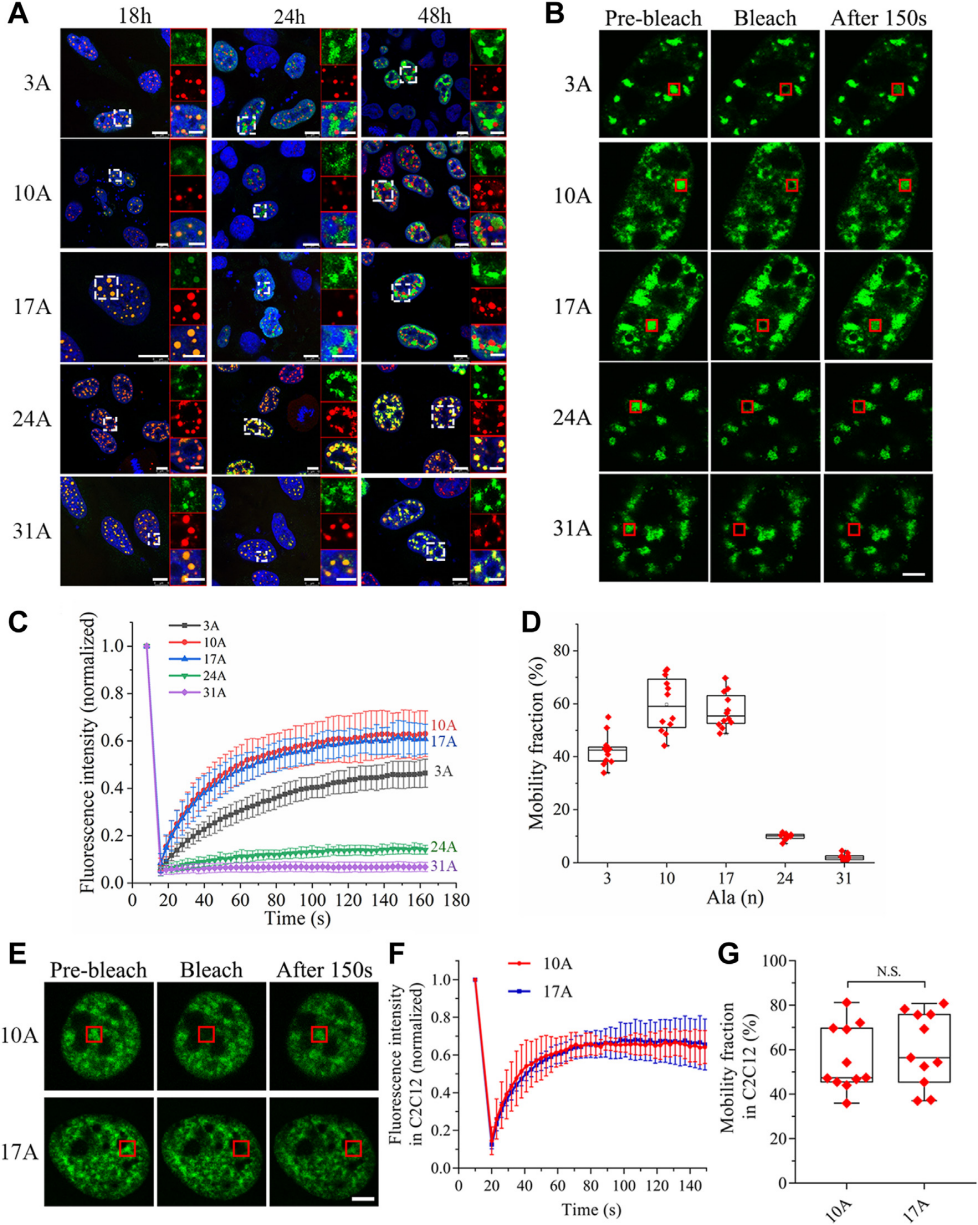
**Ala expansion reduces the mobility of PABPN1 in nucleus.***A*, time courses of the PABPN1 variants in HeLa cells. Cells were fixed and immunostained at the 18, 24, or 48-h interval after exogenous protein expression. The FLAG-tagged PABPN1 variants were stained with an anti-FLAG antibody (*green*), SC35 was stained with anti-SC35 antibody (*red*), and nuclei were stained with Hoechst (*blue*). Scale bar represents 10 μm for main panels or 5 μm for zoom image panels. *B*, FRAP analysis of the PABPN1 variants in HeLa cells. Cells were transfected with the respective EGFP-PABPN1 plasmids, and 36 h later, photobleaching was started (0 s) on the bleach spot (*red boxes*), and the recovery process was monitored by a microscope. Scale bar represents 5 μm. *C*, fluorescence recovery profiles after photobleaching. The fluorescence intensity at a bleach spot shown in (*B*) was normalized to the mean intensity of the spot before photobleaching and plotted over the recovery time. Data are represented as mean ± SD (totally from 12 recovery curves in three independent experiments). *D*, mobility fraction after 150-s recovery. Data are shown with boxplot (totally 12 data points in three independent experiments). *E*, FRAP analysis of the PABPN1 variants in C2C12 cells. C2C12 cells were transfected with the respective EGFP-PABPN1 variants, and 36 h later, photobleaching was started (0 s) on the bleach spot (*red box*), and the recovery process was monitored by a microscope. Scale bar represents 5 μm. *F*, fluorescence recovery profiles after photobleaching. The fluorescence intensity at a bleach spot shown in (*E*) was normalized to the mean intensity of the spot before photobleaching and plotted over the recovery time. Data are represented as mean ± SD (totally from 11 recovery curves in three independent experiments). *G*, mobility fraction after 150-s recovery. Data are shown with boxplot (totally 11 data points in three independent experiments, *t* test; N.S., no significance). Ala, alanine; FRAP, fluorescence recovery after photobleaching; PABPN1, Poly(A)-binding protein nuclear 1.

Previous studies have shown that nuclear speckles are dynamic or mobile structures and their components can exchange continuously with the nucleoplasm and other nuclear locations (9). To investigate the biomolecular behaviors of the PABPN1 puncta, we monitored motions of the nuclear puncta with microscopic imaging. FLAG-tagged PABPN1-10A and its variants were transiently expressed in HeLa cells, then the images were obtained at different intervals. At the 18-h point, all PABPN1 species formed small dotted and roughly round-shaped puncta that were colocalized with SC35 (Fig. 2*A*), suggesting that these proteins were incorporated into speckles or aggregates within this period. Surprisingly, after culture for 24 to 48 h, PABPN1-10A as well as the 3A and 17A variants exhibited liquid-like features that the small puncta could further fuse into larger and more irregular-shaped accumulates. Moreover, the PABPN1 molecules were disassociated with SC35 and redistributed into the nucleoplasm. It indicates that, with overexpression of PABPN1, the fusion of puncta and their component exchanges are concentration-dependent. However, the 24A and 31A variants still retained their small and round-shaped puncta colocalized with SC35, suggesting that Ala expansion enhances the aggregation propensity of PABPN1 and correspondingly reduces its punctum mobility in nucleus.

To further investigate the dynamics of PABPN1 in various puncta, we modified the constructs by fusing with EGFP at the N-terminus (EGFP-PABPN1), transiently expressed these proteins in HeLa cells, and performed fluorescence recovery after photobleaching (FRAP) experiment (38). As a result, PABPN1-10A and 17A in the nuclear puncta exhibited fast recovery after bleaching (Fig. 2*B*), indicating a feature of liquid-like droplets or speckles that experience dynamic biomolecular exchanges with the environment. With the expansion of Ala stretch, the punctum mobility was decreased dramatically (Fig. 2, *C* and *D*). The 24A variant showed a slower and incomplete recovery after bleaching, while the 31A exhibited almost no recovery that appeared to form solid-like aggregates in nucleus (Fig. 2, *B* and *C*). Unexpectedly, the 3A variant presented a reduced recovery and a small mobility fraction, as compared with the WT (10A) (Fig. 2, *C* and *D*), suggesting that appropriate Ala repeats are essential for PABPN1 to maintain its biophysical properties of liquid-like droplets. Taken together, our data support the notion that normal PABPN1 forms liquid-like nuclear speckles and Ala expansion drives the protein phase transition to solid-like aggregates that exhibit reduced biomolecular mobilities.

Since the insoluble PABPN1-17A aggregates were supposed to be relevant to the pathogenesis of a muscle-specific disease, we compared the dynamics of PABN1-10A and PABPN1-17A in C2C12 cells. The EGFP-PABPN1-10A or 17A plasmid was transfected into C2C12 cells, then 36 h later, the FRAP experiment was performed. As in the case in HeLa cells, both the PABPN1-10A and PABPN1-17A puncta exhibited fast recovery after bleaching (Fig. 2, *E* and *F*), and there was no significant difference in their mobility factions (Fig. 2*G*). This result implies that PABPN1-17A does not form solid-like aggregates but assembles into nuclear speckles similar with the WT (10A) in cultured C2C12 cells.

### RNA binding is involved in speckle formation but not in phase transition to aggregates

**Figure 3:**
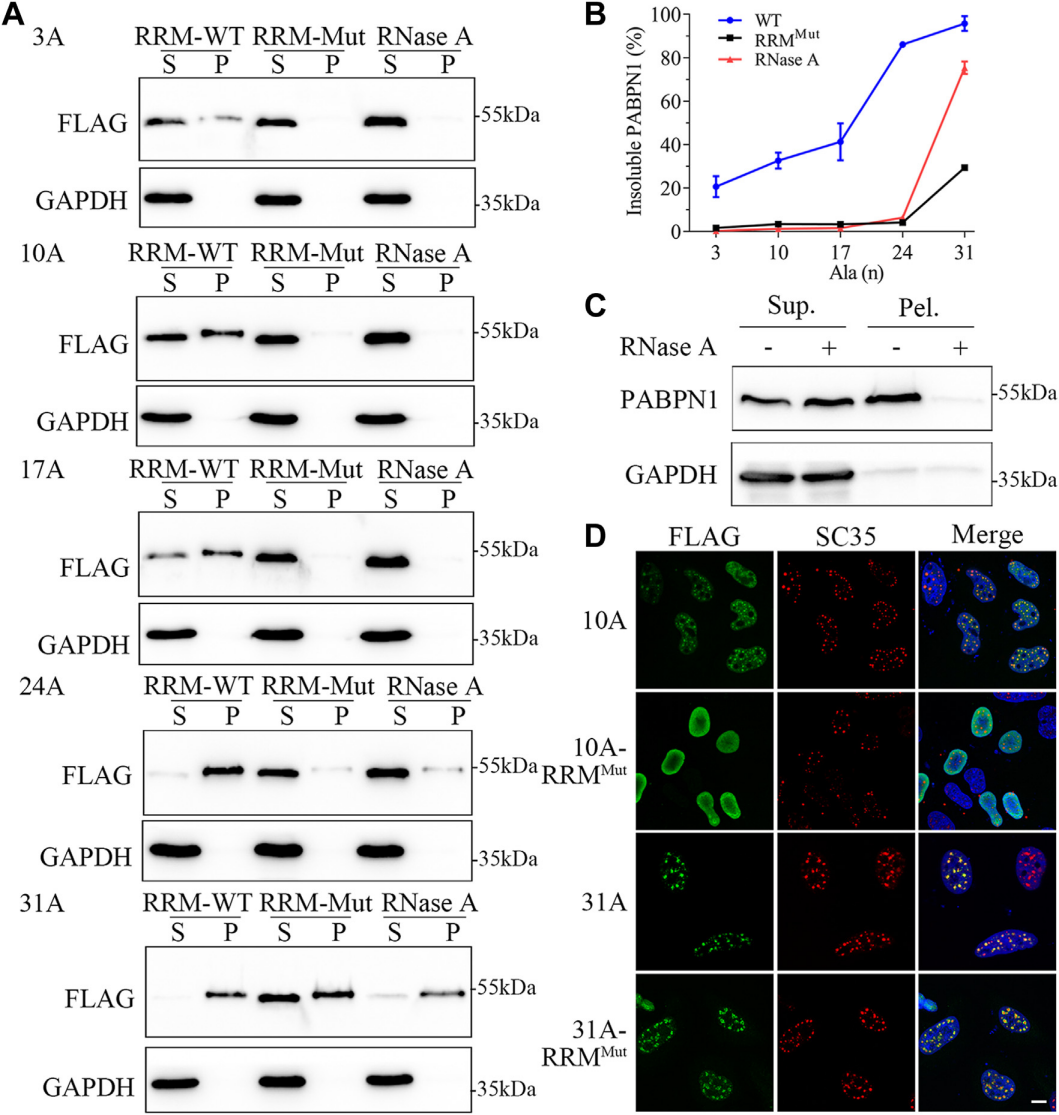
**RNA is involved in speckle formation of PABPN1.***A*, S/P fractionation analysis. FLAG-tagged PABPN1 variants and their RRM mutants were overexpressed in HeLa cells, and after 48-h culture, the cell lysates were subjected to S/P fractionation. RNase-A treatments were included in the RRM-intact species. *B*, quantification of the insoluble PABPN1 in (*A*). F_P_ (%) = P/(2S + P)∗100%. Data are shown as mean ± SD (n = 3). *C*, S/P fractionation analysis of endogenous PABPN1 with RNase-A treatment. *D*, immunofluorescence imaging of the RRM mutants of PABPN1 (10A, 31A) in HeLa cells. Cells were transfected with respective PABPN1 species, and 48 h later, the cells were fixed and immunostained with anti-FLAG and anti-SC35 antibodies. FLAG for PABPN1 variants, *green*; SC35 for nuclear speckles, *red*; Hoechst for nuclei, *blue*. Scale bar represents 10 μm. PABPN1, Poly(A)-binding protein nuclear 1; RRM, RNA-recognition motif; S/P, supernatant/pellet.

PABPN1 was previously reported as an RBP included in nuclear speckles (7). We asked whether RNA plays a function in its speckle assembly or aggregate formation. To address this issue, we applied an RNA binding–deficient mutant of PABPN1 and RNase treatment during the S/P fractionation experiments that can remove RNA from the cell lysates (39). It was reported that mutation of the aromatic residues in the RRM domains can effectively attenuate the RNA-binding abilities of PABPN1 and TDP-43 (7, 40). Sequence alignment suggested that the conserved aromatic residues (F215, Y217) in the RRM domain of PABPN1 are important to its RNA binding (Fig. S2), so we introduced a double-point mutation (F215A/Y217A) in the RRM domain of PABPN1 for the following experiments. As a result, RRM mutation strongly reduced their insoluble fractions compared to the respective WT one (RRM^WT^) (Fig. 3, *A* and *B*), except for PABPN1-31A that could only cause a small extent of reduction. Similar data were also obtained from RNase-A treatment that decreased the insoluble fractions dramatically (Fig. 3, *A* and *B*). We also confirmed the observation in endogenous PABPN1 that was thought to form nuclear speckles. As expected, the insoluble PABPN1 in pellet fraction was very sensitive to RNase-A treatment (Fig. 3*C*). Since RNase A may not digest poly(A) RNA efficiently, we also repeated the experiment by using RNase R, a nuclease that is able to cleave poly(A) tails of mRNA (41, 42). Similar to the result from RNase-A treatment, the insoluble fractions of PABPN1 variants (10A, 17A, and 31A) were significantly reduced upon RNase-R treatment (Fig. S3), while that of the endogenous PABPN1 was decreased as well. Thus, the RNA-dependent propensity of PABPN1 punctum formation decreases to some extent with the Ala expansion. PABPN1-3A, 10A, and 17A form nuclear speckles in an RNA-dependent manner, while PABPN1-31A can form RNase-resistant aggregates caused by Ala expansion. In this regard, PABPN1-24A is likely to stay in a phase boundary that partly forms aggregates.

Next, we performed immunofluorescence imaging for the RRM mutants transiently expressed in HeLa cells. The images showed that the RRM mutant of 10A (PABPN1-10A-RRM^Mut^) was completely dispersed in nucleus as compared with the WT that forms nuclear speckles (Fig. 3*D*), suggesting that RNA binding is essential to nuclear speckle formation. However, the RRM mutant of 31A (PABPN1-31A-RRM^Mut^) still retained the punctum structures in nucleus similar with the corresponding 31A form and also exhibited colocalization with SC35 (Fig. 3*D*), suggesting that PABPN1-31A forms nuclear aggregates independent on RNA binding. Collectively, WT PABPN1 forms dynamic nuclear speckles, while Ala expansion (>17A ∼ 24A) may trigger PABPN1 phase transition to solid-like aggregates. RNA plays an important role in facilitating PABPN1 assembly into nuclear speckles, but it is not indispensable for aggregation.

### Poly(A) nucleotide is essential to mediating early-stage condensation of PABPN1

**Figure 4:**
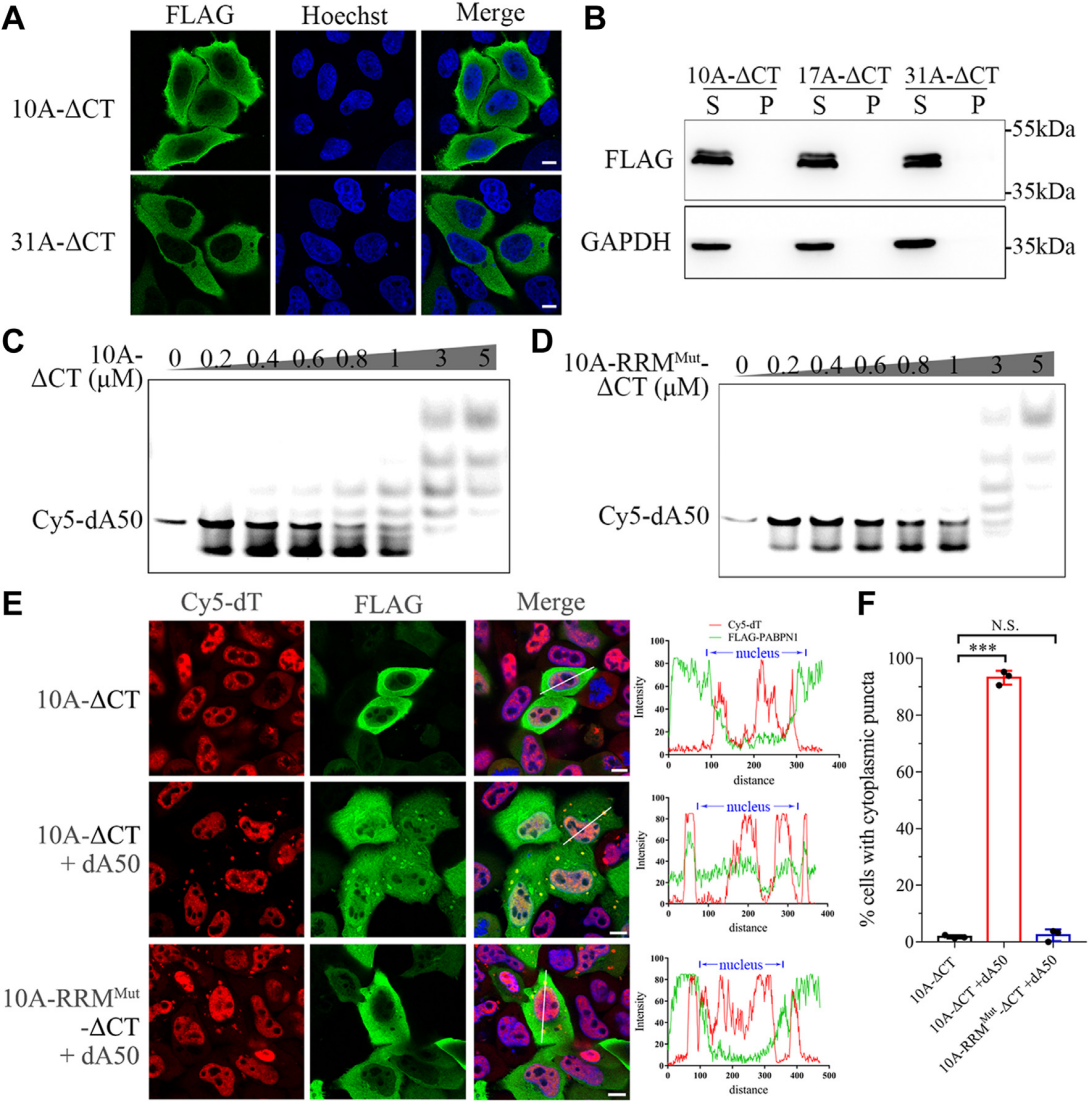
**Poly(A) nucleotide drives condensation of cytoplasmic PABPN1.***A*, immunofluorescence imaging of the NLS-deleted (ΔCT) variants of PABPN1. FLAG-tagged PABPN1-10A-ΔCT or 31A-ΔCT was transfected into HeLa cells, and 48 h later, the cells were subjected to immunofluorescence imaging. FLAG for PABPN1 variants, *green*; Hoechst for nuclei, *blue*. Scale bar represents 10 μm. *B*, S/P fractionation analysis of the ΔCT species. *C* and *D*, EMSA for deoxy-oligonucleotide dA50 binding to the PABPN1 variants, 10A-ΔCT (*C*) or 10A-RRM^Mut^-ΔCT (*D*). Cy5-labeled dA50 (2 nM) was incubated with increasing amount of the PABPN1 variants (0–5 μM). *E*, FISH combined with immunofluorescence imaging of the cytoplasmic condensates in HeLa cells. Cells were cotransfected with FLAG-PABPN1-10A-ΔCT or its RRM mutant and dA50, and 24 h later, the cells were fixed and immunostained. The PABPN1 variants were stained with an anti-FLAG antibody (*green*); dA50 was stained with Cy5-dT (*red*), and nuclei were stained with Hoechst (*blue*). Scale bar represents 10 μm. *Right*: colocalization analysis of the fluorescence signals. *F*, quantitative analysis of the cells with cytoplasmic puncta. The percent of the cells with cytoplasmic puncta was analyzed (totally 257 cells in three independent experiments) and presented with mean ± SD (*t* test; ∗∗∗*p* < 0.001; N.S., no significance). PABPN1, Poly(A)-binding protein nuclear 1; RRM, RNA-recognition motif; NLS, nuclear localization signal; S/P, supernatant/pellet.

PABPN1-31A can form RNase-resistant aggregates, but there is still a large difference between RNase treatment and RRM mutation in PABPN1-31A (Fig. 3*B*). It may raise a question whether RNA plays a role in the early-stage condensation of PABPN1-31A because the PABPN1-31A aggregates have already formed before RNase treatment. As known, PABPN1 has a high affinity to poly(A) tails, and poly(A) RNAs mainly exist in the nucleus. Some mutant RBPs, such as TDP-43, FUS, and hnRNP A1, have been reported to extensively form cytoplasmic aggregates or inclusions (20, 27). However, we have observed that the Ala-expanded variants of PABPN1 assembled into speckles or aggregates but remained in the nuclei. To clarify this inconsistency, we generated the NLS-deleted constructs of PABPN1 (PABPN1-ΔCT) and transiently expressed them in HeLa cells. Immunofluorescence imaging showed that both forms of PABPN1 (10A-ΔCT, 31A-ΔCT) could not assemble into puncta but diffusely distributed in cytoplasm (Fig. 4*A*), which was different from their nuclear forms assembled into either speckles or aggregates obviously (Fig. 1*F*). Consistent with this observation, S/P fractionation showed that neither 10A nor 31A could be detected in the insoluble pellet fraction (Fig. 4*B*). These data suggest that Ala expansion only is not sufficient to trigger aggregate formation of PABPN1 in cytoplasm, but some other factors in the nuclear environment may be contributable to the aggregation. Since RNA is an important driver of early-stage condensation of PABPN1 (Fig. 3*D*) and poly(A) mRNA is abundant in nucleus, we speculate that the concentration of the poly(A)-containing RNAs (*e.g.*, mRNA) in cytoplasm is too low to trigger PABPN1 self-assembly into condensates and aggregates thereafter.

It is known that PABPN1 binds with high affinity to the poly(A) tails of mRNAs (43). To test this hypothesis, we synthesized a 50-nt deoxy-adenoside oligomer (dA50) to mimic poly(A) nucleotide and transfected it into HeLa cells to generate an environment of high concentration of poly(A) mRNA in cytoplasm. At first, EMSA assay confirmed that 10A-ΔCT bound to dA50 efficiently *in vitro* (Fig. 4*C*), whereas RRM mutation weakened their association considerably (Fig. 4*D*). Then, dA50 was transiently transfected with 10A-ΔCT or its RRM mutant into HeLa cells, and the cells were subjected to FISH. As expected, upon addition of dA50 into cytoplasm, the number of the 10A-ΔCT puncta was significantly increased in the cytoplasm (Fig. 4*E*). About 93.2% of the dA50-positive cells were detected with cytoplasmic puncta when transfected with 10A-ΔCT (Fig. 4*F*). Meanwhile, colocalization of dA50 with the cytoplasmic puncta suggests that dA50 was successfully delivered into the cytoplasm and bound to the cytoplasmic form of PABPN1. Conversely, the RRM mutant of 10A-ΔCT with deficient dA50 binding remained completely dispersed and could not form any visible puncta in cytoplasm even in the presence of dA50 (Fig. 4, *E* and *F*). These data imply that, in the early-stage condensation of PABPN1, poly(A) mRNA is a determinant in formation of the puncta. The concentration of poly(A) mRNA in cytoplasm is not enough to drive PABPN1 condensation nor aggregation, which convincingly explain the observation that the Ala-expanded variants can only self-assemble into nuclear aggregates.

### Poly(A) nucleotide modulates PS of PABPN1 *in vitro*

**Figure 5:**
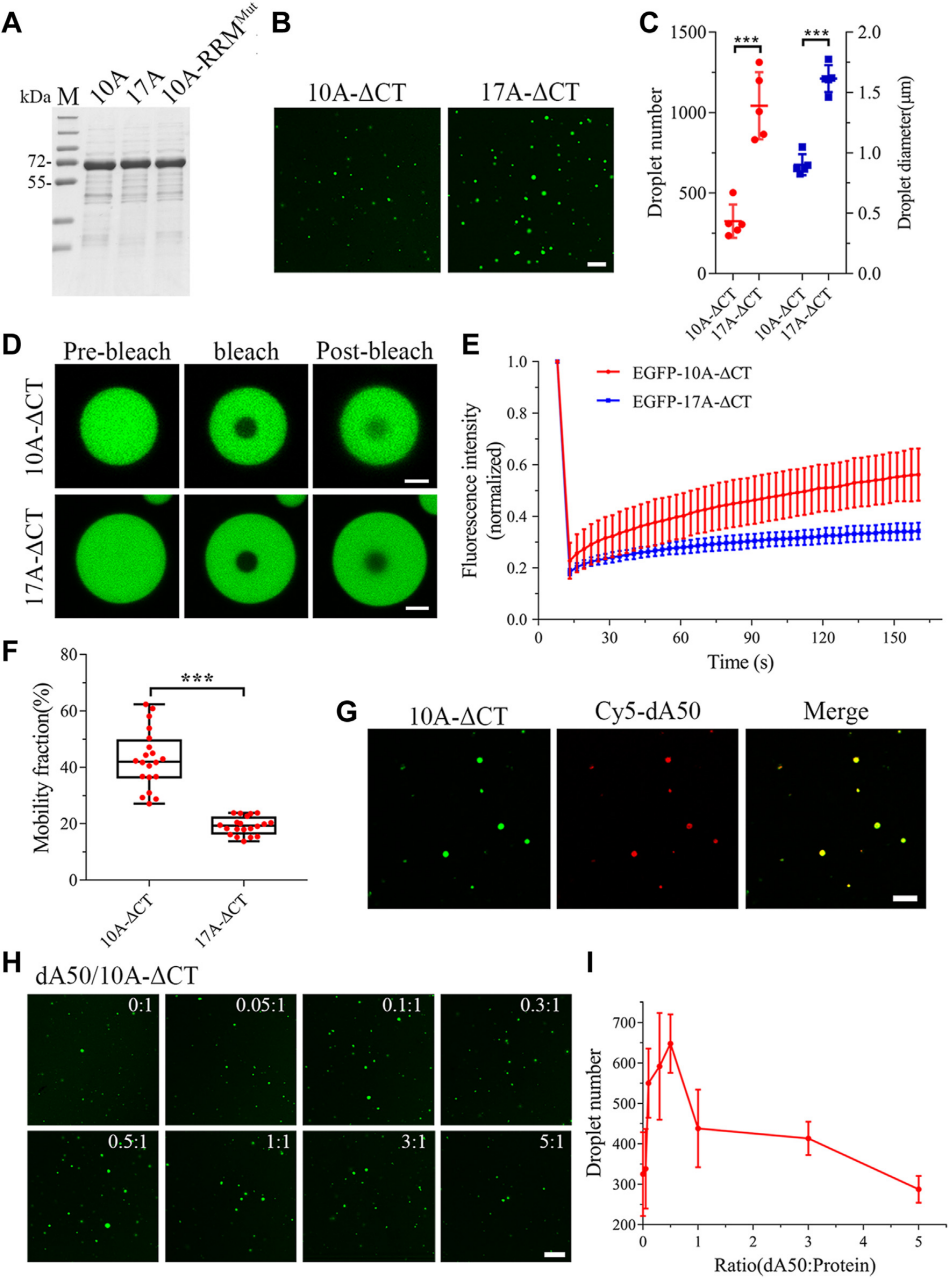
**Poly(A) nucleotide modulates droplet condensation of PABPN1 *in vitro*.***A*, purification of the recombinant PABPN1 proteins. Each 5 μM of EGFP-tagged PABPN1-10A-ΔCT, 17A-ΔCT, and 10A-RRM^Mut^-ΔCT was loaded in SDS-PAGE gels with Coomassie blue staining. *B*, *in-vitro* droplet assay for the EGFP-tagged PABPN1-10A-ΔCT and 17A-ΔCT variants. Each 5-μM protein in 120 mM NaCl, 10% PEG was incubated for 30 min and imaged with confocal microscopy. Scale bar represents 5 μm. *C*, quantification of the droplet number (*left*, *red*) and diameter (*right*, *blue*) of the puncta in (*B*). Data are shown as mean ± SD (n = 5, *t* test); ∗∗∗*p* < 0.001. *D*, FRAP analysis of the EGFP-tagged PABPN1-10A-ΔCT and 17A-ΔCT variants *in vitro*. Each 20-μM protein in 120 mM NaCl, 10% PEG was incubated for 30 min, and FRAP was started and the recovery was recorded. Scale bar represents 2 μm. *E*, fluorescence recovery profiles for the ΔCT species after photobleaching. The fluorescence intensity at a bleach spot shown in (*D*) was normalized to the mean intensity of the spot before bleaching and represented as mean ± SD (totally 20 recovery curves in three independent experiments). *F*, mobility fraction for the ΔCT species after the recovery time of 150 s. Data are shown with boxplot (totally 20 areas in three independent experiments). ∗∗∗*p* < 0.001. *G*, colocalization of PABPN1-10A-ΔCT with dA50 in droplets. Five micromolar of EGFP-PABPN1-10A-ΔCT (*green*) was incubated with 1.5 μM of dA50 (10% Cy5-dA50, *red*) in a solution of 120 mM NaCl and 10% PEG. Scale bar represents 10 μm. *H*, *in-vitro* droplet formation of PABPN1-10A-ΔCT with various concentrations of dA50. Five micromolars of EGFP-PABPN1-10A-ΔCT (*green*) was incubated with indicated concentration of dA50 in a solution of 120 mM NaCl and 10% PEG for 30 min and imaged with confocal microscopy. Scale bar represents 10 μm. *I*, quantification of the droplet number in (*H*). Data are shown as mean ± SD (n = 5). FRAP, fluorescence recovery after photobleaching; PABPN1, Poly(A)-binding protein nuclear 1; RRM, RNA-recognition motif.

Since normal PABPN1 forms RNase-sensitive and droplet-like nuclear speckles and poly(A) nucleotide has important contribution to the phase behavior of PABPN1 in cells, we then ask how poly(A) nucleotide modulates PS of PABPN1 *in vitro*. We generated three recombinant PABPN1 constructs with NLS deletion and EGFP fusion, namely EGFP-PABPN1-10A-ΔCT and its 17A and RRM variants. We purified these proteins (Fig. 5*A*) and performed PS assay in the absence of RNA. Firstly, we examined the PS behaviors of 10A-ΔCT and 17A-ΔCT and found that both could form liquid-like droplets *in vitro* (Fig. 5, *B* and *C*), as in the case of full-length PABPN1 (44). The number and average diameter of the droplets in 17A-ΔCT were much larger than those in 10A-ΔCT at the same protein concentration (5 μM), indicating that the 17A variant of PABPN1 possesses a stronger ability to phase separate into droplets than the WT. Moreover, FRAP experiment showed that Ala expansion reduced the internal mobility of droplets to some extent (Fig. 5, *D* and *E*). The mobility fraction of 17A-ΔCT was only 19.3%, compared with 42.9% of 10A-ΔCT (Fig. 5*F*). Next, we examined whether dA50 affects droplet formation of PABPN1 *in vitro*. The droplets formed by 10A-ΔCT were colocalized with the fluorescent-labeled dA50 (Fig. 5*G*). With the increase of dA50, it could promote droplet formation of 10A-ΔCT till a maximum at the dA50/10A-ΔCT ratio of 0.5: 1, but the promoting effect was alleviated at the higher doses of dA50 (Fig. 5, *H* and *I*). It seemed that the optimal ratio of dA50/10A-ΔCT was approximately 1: 2, that is, at this stage, a dA50 chain may bind two protein molecules stoichiometrically for effectively promoting PS of PABPN1. In contrast, droplet formation of the RRM mutant (10A-RRM^mut^-ΔCT) was not affected remarkably by addition of dA50 (Fig. S4, *A* and *C*), due to its deficiency in dA50 binding (Fig. 4*D*). On the other hand, dA50 could also promote droplet formation of 17A-ΔCT considerably to a plateau at larger dA50/17A-ΔCT ratios (>1: 1) (Fig. S4, *B* and *C*), being consistent with the observation from EMSA that 17A-ΔCT bound to dA50 more weakly than 10A-ΔCT (Fig. S4*D*). Thus, the *in vitro* study substantiates the observation in cells that poly(A) nucleotide is involved in PS of PABPN1.

### The PABPN1 aggregates sequester CFIm25 through specific RNA binding

**Figure 6:**
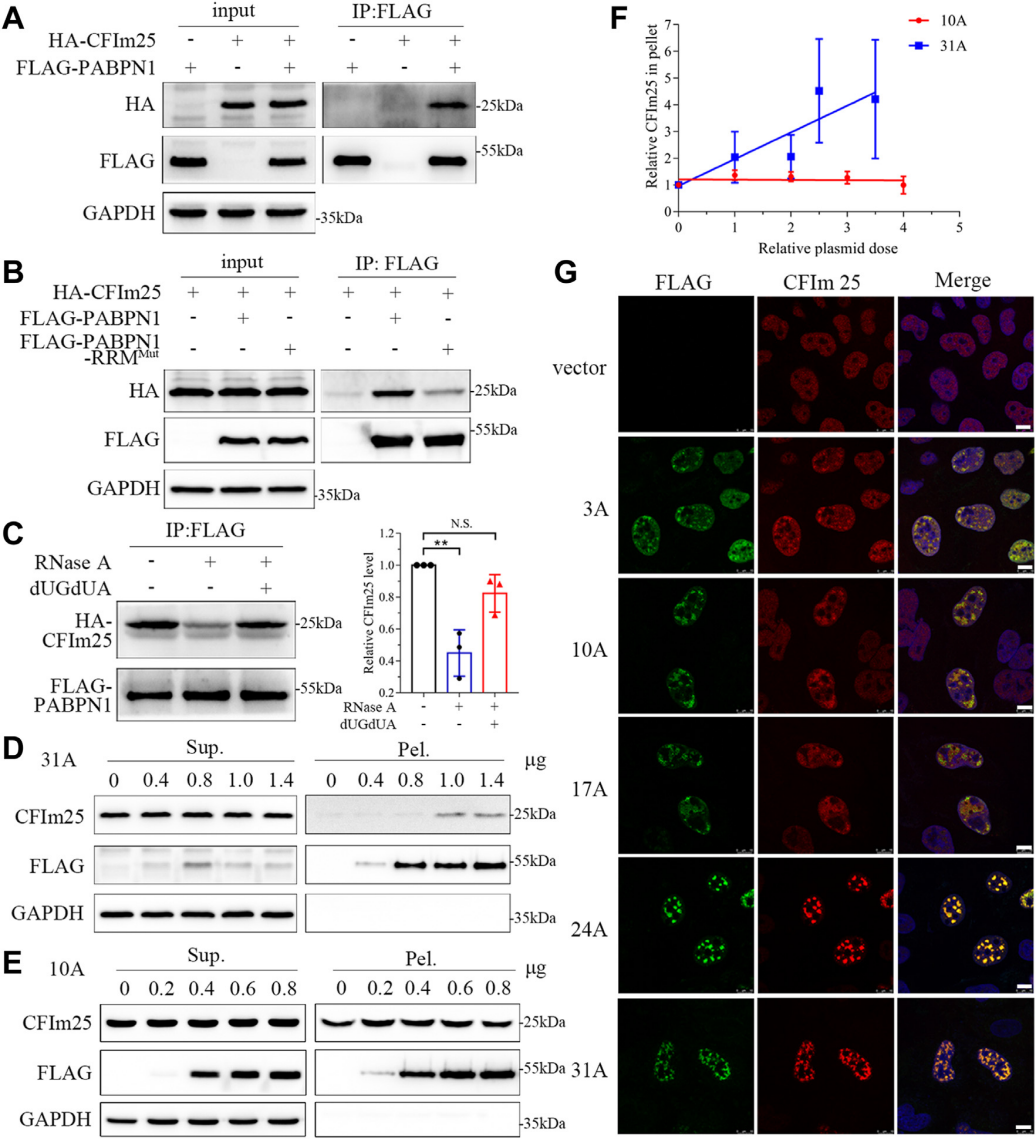
**The PABPN1 aggregates sequester CFIm25 through sequence-specific RNA.***A* and *B*, Co-IP analysis of the interaction of WT PABPN1 (*A*) or PABPN1-RRM^Mut^ (*B*) with CFIm25. HEK 293T cells were transfected with the indicated plasmids, and 48 h later, the cell lysates were subjected to immunoprecipitation with an anti-FLAG antibody. *C*, Co-IP analysis under the conditions of RNase A and/or ssDNA treatments. RNase A and/or dUGdUA ssDNA were added into the cell lysates before immunoprecipitation. The *right panel* shows the relative CFIm25 levels, and the data are presented as mean ± SD (n = 3). ∗∗*p* < 0.01. *D* and *E*, dose-dependent experiment for CFIm25 sequestered by PABPN1-31A (*D*) or PABPN1-10A (*E*). HEK 293T cells were transfected with the FLAG-tagged PABPN1 plasmid at an increasing dose, and 48 h later, the cell lysates were then subjected to S/P fractionation. *F*, quantification of CFIm25 in the pellet fraction in (*D*) and (*E*) sequestered by the PABPN1 aggregates. Data were normalized with that of the vector control and presented with mean ± SD (n = 3). *G*, immunofluorescence imaging of the PABPN1 variants with endogenous CFIm25. HeLa cells were transfected with the FLAG-tagged PABPN1 variants, and after 48-h culture, the cells were fixed and immunostained with anti-FLAG (*green*) and anti-CFIm25 (*red*) antibodies. Nuclei were stained with Hoechst (*blue*). Scale bar represents 10 μm. CFIm25, 25-kDa component of the mammalian cleavage factor I complex; co-IP, co-immunoprecipitation; N. S. no significance; PABPN1, Poly(A)-binding protein nuclear 1; RRM, RNA-recognition motif; S/P, supernatant/pellet.

Since Ala expansion can enhance aggregate formation of PABPN1, we next inquire whether PABPN1 aggregation leads to sequestration of the components of pre-mRNA 3′-UTR processing complex and thereby causes cellular toxicity. As reported, PABPN1 could interact with CFIm25 directly (19); we then re-examined their interactions by co-immunoprecipitation assay. The data showed that PABPN1 could associate with CFIm25 (Fig. 6*A*). Interestingly, this association between PABPN1 and CFIm25 was obviously attenuated by mutation in the RRM domain of PABPN1 (Fig. 6*B*) or RNase-A treatment (Fig. 6*C*, middle lane), suggesting that RNA plays an important role in mediating the association between these two RBPs. To further verify the specific RNA sequences that assist in their association, we synthesized two pieces of ssDNA, namely TGTA and dUGdUA (Table S3), to mimic the corresponding RNA that simultaneously binds to both PABPN1 and CFIm25 (43, 45) and performed co-immunoprecipitation experiments under the conditions of RNase-A plus ssDNA treatment (39). The result showed that the dUGdUA sequence could largely rescue the attenuating effect of RNase-A treatment (Fig. 6*C*, right lane), whereas TGTA ssDNA could not (Fig. S5*A*). These data suggest that PABPN1 interacts with CFIm25 indirectly and their association is dependent on sequence-specific RNAs.

We then examined whether the PABPN1 speckles or aggregates sequester endogenous CFIm25. Different doses of FLAG-tagged PABPN1-10A or 31A were transfected into HEK 293T cells, and then S/P fractionation was performed to detect endogenous CFIm25 precipitated in the pellet fraction. The result showed that the amount of CFIm25 in pellet fraction was significantly increased with the increasing dose of PABPN1-31A (Fig. 6, *D* and *F*). As a contrast, the CFIm25 amount in insoluble fraction was almost unchanged with the increase of PABPN1-10A (Fig. 6, *E* and *F*). It strongly indicates that the aggregates formed by PABPN1-31A drive the endogenous CFIm25 protein to be enriched into the insoluble fraction, but the dynamic speckles of WT PABPN1 do not. In addition, immunofluorescence imaging showed that endogenous CFIm25 was colocalized with all species of PABPN1 (Fig. 6*G*), further demonstrating the association of PABPN1 with CFIm25 with the assistance of RNA. However, only the 24A and 31A variants could form solid-like aggregates and sequester CFIm25 into the round-shaped and compact puncta, as compared with the other three species (3A, 10A, and 17A) that only caused partial distribution with CFIm25. Similar result was also obtained from exogenous expression of CFIm25 in which CFIm25 could be sequestered and colocalized with the aggregates formed by PABPN1-24A or 31A (Fig. S5*B*). However, the CFIm25 protein was still diffusely distributed in nucleus during overexpression of PABPN1-3A, 10A, or 17A, albeit they could form nuclear speckles. Together, we conclude that the PABPN1 aggregates, but not the speckles, sequester CFIm25 into nuclear aggregates depending on RNA-mediated protein association.

### The PABPN1 aggregates interfere with distal PAS usage through depriving the availability of CFIm25

**Figure 7:**
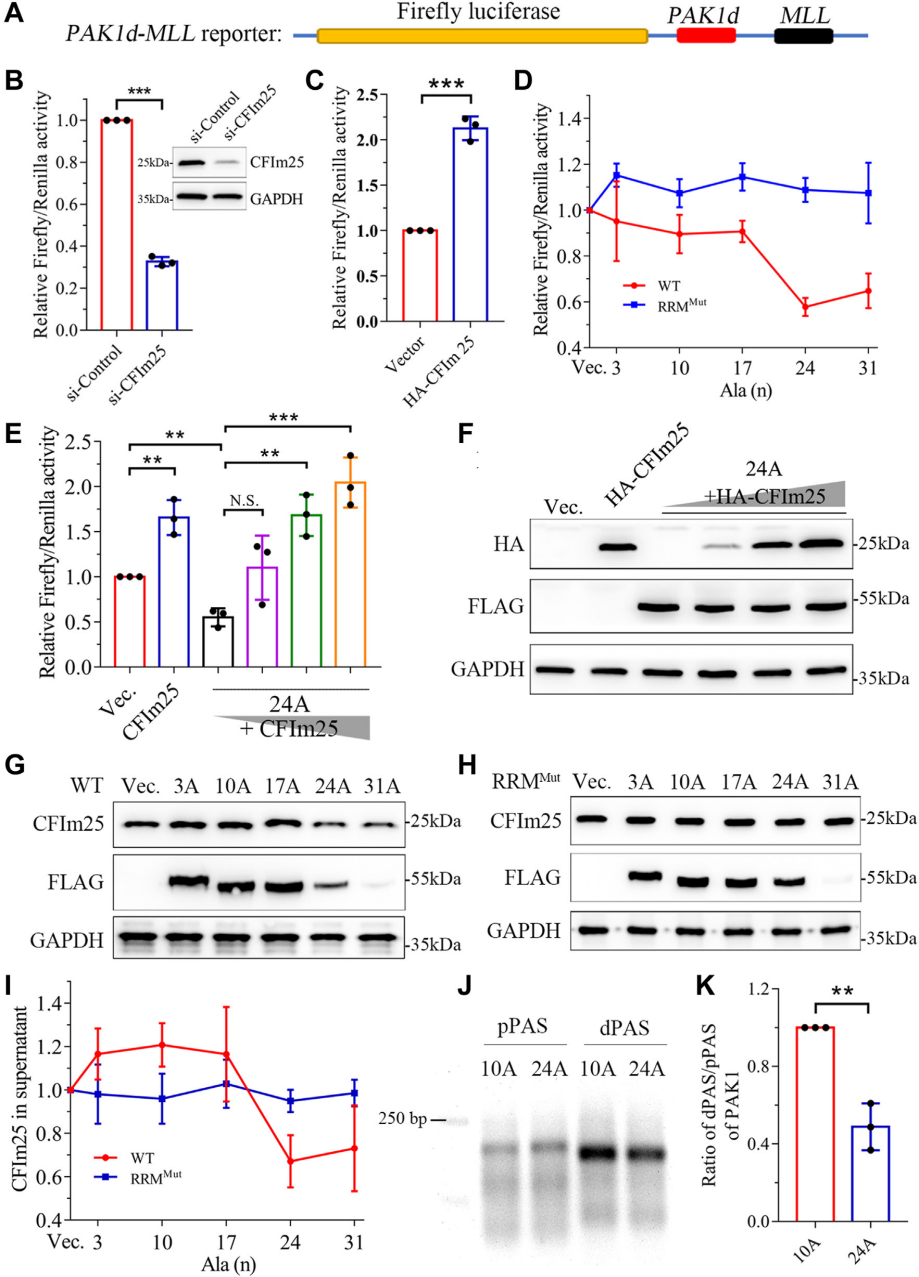
**The PABPN1 aggregates alter the CFIm25 function in APA.***A*, schematic diagram of the reporter plasmid. The *PAK1d-MLL* reporter plasmid was constructed by linking the DNA sequences of *Firefly* luciferase and distal *PAK1* and *MLL* in the vector of pmirgol1. The sequence encoding *Renilla* luciferase was also included in the plasmid. *B*, relative Firefly/Renilla luciferase activity of the *PAK1d-MLL* reporter upon knockdown of CFIm25. The reporter and siRNA were transfected into HEK 293T cells, and 48 h later, the fluorescence data were recorded. The inset shows knockdown of CFIm25 by siRNA interference. Data are shown as mean ± SD (n = 3, *t* test). ∗∗∗*p* < 0.001. *C*, relative luciferase activity upon overexpression of CFIm25 in HEK 293T cells. Data are shown as mean ± SD (n = 3, *t* test). ∗∗∗*p* < 0.001. *D*, effect of Ala expansion in PABPN1 or its RRM mutant on the relative luciferase activity. HEK 293T cells were transfected with the reporter plasmid and various FLAG-tagged PABPN1 variants (RRM^WT^) or their RRM mutants (RRM^Mut^), respectively, and 48 h later, each luciferase activity was recorded and compared. Data shown were relative Firefly/Renilla luciferase activities normalized to that of the vector control and presented as mean ± SD (n = 3). *E*, the rescue effect of CFIm25 on the suppression of luciferase activity caused by PABPN1-24A. Data are shown as mean ± SD (n = 3, *t* test). ∗∗*p* < 0.01; ∗∗∗*p* < 0.001. *F*, Western blotting analysis for CFIm25 (*E*) in the supernatant fraction. *G* and *H*, Western blotting analysis of soluble CFIm25 in supernatant fraction upon overexpression of the PABPN1 variants (RRM^WT^) (*G*) or their RRM mutants (RRM^Mut^) (*H*) in HEK 293T cells. *I*, quantification of the soluble fraction of CFIm25 in supernatant. Data were from (*G*) and (*H*), normalized to that of vector, and shown as mean ± SD (n = 3). *J*, detection of the distal and proximal PAS of *PAK1* by RT-PCR. HEK 293T cells were transfected with PABPN1-10A or PABPN1-24A, and 48 h later, the total RNA was isolated and its cDNA was synthesized by RHAPA after PCR amplification using primers for dPAS and pPAS of *PAK1*. The PCR products were analyzed by 1.5% agarose gel electrophoresis. *K*, quantification of the dPAS and pPAS levels of *PAK1*. Data are represented with the ratio of dPAS/pPAS and shown as mean ± SD (n = 3). ∗∗*p* < 0.01. Ala, alanine; APA, alternative polyadenylation; CFIm25, 25-kDa component of the mammalian cleavage factor I complex; MLL, mixed leukemia lymphoma; N.S., not significant; PABPN1, Poly(A)-binding protein nuclear 1; PAS, polyadenylation site; RHAPA, RNase H alternative polyadenylation assay; RRM, RNA-recognition motif.

Previous research has demonstrated that CFIm25 contributes to the APA function in pre-mRNA 3′-UTR processing (46). CFIm25 binds specifically to the UGUA repeat sequences that are mainly prevalent in the distal PAS of pre-mRNA (47) and thereby stimulates the usage of distal PAS. In the presence of CFIm25, it has a priority to use distal PAS, while knock-down of CFIm25 increases the usage of proximal PAS (46). Given our observation that the Ala-expanded PABPN1 could sequester CFIm25 into aggregates (Fig. 6), we proposed that this would deprive the availability of active CFIm25. As *PAK1* is a downstream target gene of CFIm25 (48), we designed a dual luciferase reporter, namely *PAK1d-MLL*. The reporter consists of an ORF of Firefly luciferase followed by the distal PAS region of *PAK1* 3′-UTR and a minimized mixed leukemia lymphoma (*MLL*) 3′-UTR (49), along with an independent Renilla luciferase element as a control (Fig. 7*A*). We expected that the repressive effect on the expression of Firefly luciferase by *MLL* 3′-UTR would be alleviated due to the early cleavage mediated by *PAK1* dPAS in the presence of CFIm25. To validate the system, the reporter plasmid was transfected into the CFIm25-knockdown cells, and then the luciferase expression was recorded and compared. As expected, knockdown of CFIm25 strongly reduced the ratio of Firefly/Renilla activity as to about 3-fold (Fig. 7*B*). Moreover, overexpression of CFIm25 remarkably increased the ratio compared with the control (Fig. 7*C*). This indicates that the reporter system is suitable to monitoring the functional change of CFIm25 caused by Ala expansion of PABPN1.

To examine the function of CFIm25 in response to Ala expansion of PABPN1, various species of PABPN1 were tested and the luciferase activities were recorded and compared. The result showed that the Firefly/Renilla ratio was obviously decreased in the cells overexpressing Ala-expanded variants, PABPN1-24A or 31A (Fig. 7*D*). In contrast, the ratio remained almost unchanged with the expansion of Ala stretch when the cells were overexpressing the respective RRM mutants, suggesting that the functional loss of CFIm25 by Ala expansion in PABPN1 is related to RNA binding. Thus, the three species (3A, 10A, and 17A) that form relatively dynamic speckles may have little impact on the function of CFIm25, whereas the 24A and 31A variants that can form immobile solid-like aggregates may result in the loss of function of CFIm25. Further experiment showed that the repressive effect of the 24A variant could be restored by the overexpression of CFIm25 (Fig. 7, *E* and *F*), confirming that disruption of the CFIm25 function was caused by sequestering CFIm25 into the nuclear aggregates of PABPN1.

We next performed S/P fractionation to detect the soluble fraction of endogenous CFIm25 that may be available for APA in the presence of various PABPN1 species. Consistent with the data from luciferase reporter assay, the soluble fraction of CFIm25 was reduced significantly in the cells overexpressing PABPN1-24A or 31A, as compared to those overexpressing PABPN1-3A, 10A, or 17A (Fig. 7, *G* and *I*). However, the soluble fraction of CFIm25 maintained almost constant when the cells were overexpressing the respective RRM mutants (Fig. 7, *H* and *I*). Note that the 31A variant was sparsely detected in supernatant due to the formation of insoluble aggregates. Thus, the poly(A)-containing RNAs (*e.g.*, mRNA) with specific sequences contribute to the sequestration of CFIm25 by the PABPN1 aggregates. We also detected the usage of distal PAS of the endogenous *PAK1* gene by RT-PCR assay (48). The data showed that the distal PAS usage of *PAK1* was dramatically decreased in the cells overexpressing PABPN1-24A as compared with PABPN1-10A (Fig. 7, *J* and *K*), indicating that the PABPN1-24A aggregates significantly suppress the function of CFIm25 in APA. Together, all the data demonstrate that Ala-expanded PABPN1 forms nuclear aggregates and sequesters CFIm25 *via* sequence-specific poly(A) mRNA, which consequently deprives the availability of soluble active CFIm25 and impairs its function in APA.

## Discussion

### Ala expansion leads to phase transition of PABPN1 from nuclear speckles to aggregates

It is generally acknowledged that Ala expansion of PABPN1 is associated with the pathogenesis of OPMD disease (10, 11), whereas some studies have shown that PABPN1 aggregation is independent on the Ala stretch at its N-terminus (33, 34, 35). In the early reports, the pathogenic aggregates of PABPN1 were sometimes confused with the functional nuclear speckles, due to their morphological similarities under microscopic observation (Fig. 1*F*). Even, the exogenically overexpressed proteins of PABPN1-10A and 17A have little difference in their morphologies and dynamic properties (34), raising a possibility that the puncta formed by the 17A variant are actually nuclear speckles but not aggregates in cultured cells. Some researchers have realized this problem and sought to use high concentration of salt (*e.g.*, 1 M KCl) when visualizing the puncta by optical microscopy because the PABPN1 aggregates are resistant to salt extraction (30, 31, 32). Our FRAP study has actually validated the observation that the puncta assembled by both PABPN1-10A and 17A exhibit a similar feature of droplet-like speckles in cultured cells (Fig. 2). Either shortening or expansion of the Ala stretch will reduce the molecular mobility of PABPN1, especially the 31A variant that presents a solid-like behavior. Therefore, Ala stretch may determine the dynamics or mobility of PABPN1 in nuclear speckles; an appropriate length of Ala repeats is necessary for PABPN1 to maintain its biomolecular properties of droplet-like speckles. Expansion of the Ala stretch will lead PABPN1 to undergo phase transition from dynamic nuclear speckles to solid amyloid-like aggregates.

### RNA binding is required for PABPN1 condensation

It has been well studied for several decades that some neurodegenerative diseases, such as Huntington’s and other polyglutamine (polyQ) diseases, are resulted from an unstable trinucleotide repeat expansion (50, 51). It is worth noting that PABPN1 not only possesses Ala expansion like polyQ expansion but also harbors an RRM domain–sharing characteristics with other RBPs. Recent studies have demonstrated that these RBPs tend to self-assembly and undergo PS (52, 53), which reminds us to rethink the PABPN1 proteinopathy. Distinct from polyQ proteins, aggregation of PABPN1 is not just depending on the Ala expansion but also its RNA-binding ability. The Ala stretch only is not sufficient to mediate its condensation into droplet-like speckles in cells, for the RNA binding–deficient version of PABPN1 cannot assemble into nuclear speckles. In this study, we have shown that PABPN1 undergoes PS to condensed droplets *in vitro* and the condensation process can be facilitated by binding with poly(A) nucleotide (Fig. 5). The NLS-deleted form of PABPN1 has lost its capacity of forming puncta in cytoplasm without the assistance of RNA, but it can phase-separate into cytoplasmic condensates in the presence of poly(A) nucleotide even under a non-nuclear environment (Fig. 4). However, the NLS-deleted form of PABPN1-31A is still diffusely distributed in cytoplasm, neither assembling into speckles nor forming aggregates. Thus, the poly(A)-containing RNAs especially mRNA may be involved in the early-stage condensation of PABPN1 for the speckle formation thereafter, while the Ala stretch modulates the molecular mobility of the condensates and Ala expansion drives phase transition to the less dynamic solid-like aggregates.

PABPN1 is not a protein alone with an Ala stretch; some transcription factors, for example, PHOX2B (54), also harbor an Ala stretch in their sequences. Expansion of their Ala repeats beyond a certain threshold will lead to relocalization of these nuclear proteins to the cytoplasm and formation of cytoplasmic aggregates (55). Similar events also occur in some nuclear RBPs like TDP-43, FUS, and hnRNP A1 in which mutations may result in extensive mislocalization and cytoplasmic aggregation (56, 57, 58). As an exception, the Ala-expanded PABPN1 still maintains its nuclear localization and confers with the ability to form nuclear aggregates. The multivalent interaction properties render poly(A) nucleotide a unique feature of promoting early-stage condensation of PABPN1 that tends to assemble into speckles and further form aggregates in cells (Fig. 4). On the contrary, it appears that the poly(A) nucleotide is less important to the late-stage aggregation of Ala-expanded PABPN1. RNA-binding deficiency or RNase treatment can only partially interrupt formation of the nuclear aggregates of PABPN1-31A (Fig. 3). Meanwhile, the result from poly(A) mimics is consistent with the previous viewpoint that the RNA concentration is also important for controlling PS of RBPs (25).

### Sequestration of the components interferes with pre-mRNA 3′-UTR processing

The sequestration model proposes that the protein aggregates sequester cellular interacting factors and consequently result in disease pathology (59, 60). PABPN1 has ever been supposed to interact with CFIm25 directly (19), but our study has suggested that PABPN1 associates with CFIm25 with the assistance of sequence-specific RNA. Interestingly, the dUGdUA chimera but not the TGTA repeats can restore the interaction disrupted by RNase-A treatment. A rational explanation is that CFIm25 specifically binds to the dUGdUA repeats of ssDNA or the UGUA sites of pre-mRNA (47) but not to the TGTA repeats of ssDNA. Thus, the sequence-specific mRNAs are involved in the association of PABPN1 with CFIm25. Consequently, the nuclear aggregates formed by Ala-expanded PABPN1 sequester endogenous CFIm25 mediated by sequence-specific mRNAs, as in the case of the cytoplasmic TDP-35 inclusions that sequester TDP-43 and other RBPs assisted by RNAs (39, 40). It has been reported that PABPN1 prevents utilization of proximal PAS by binding to a specific RNA region and overexpression of Ala-expanded variants can sequester endogenous PABPN1 into nuclear aggregates, leading to relieve the inhibiting effect of normal PABPN1 and a shift in APA towards the proximal site usage (4). We herein propose an alternative mechanism underlying the alteration of PAS usage due to the PABPN1 aggregates in which Ala-expanded PABPN1 forms nuclear aggregates and sequesters CFIm25 so as to deprive the availability of soluble active CFIm25 and thus to suppress the distal PAS usage in APA.

### Phase transition of PABPN1 and its disease pathology

Ala expansion of PANPN1 is generally considered to associate closely with the pathogenesis of OPMD (11, 12). PABPN1 has been detected in the intranuclear inclusions (aggregates) from OPMD patients, which are insoluble and resistant to the treatment with 1 M KCl (61). Similar observation has also been made in muscles from PABPN1-17A knock-in or overexpressed mouse (30, 31, 32). OPMD is an orphan disease and the patient specimens are rare. According to the statistics, only 0.1 ∼ 1: 100,000 occurs in the higher-incidence European population, which results in few researches on the PABPN1 aggregates (62). It is generally acknowledged that the 17A variant could form insoluble aggregates in OPMD patients or mouse models, but at least in cultured cells even in muscle cells, our data suggest that the 17A variant is similar with normal PABPN1 in phase behaviors. We have shown that the biomolecular behaviors of the 17A variant are very similar to those of the WT (10A), that is, both can assemble into dynamic nuclear speckles but cannot undergo phase transition to aggregates and sequestration of CFIm25, whether they are overexpressed in HEK 293T, HeLa, or C2C12 cells. OPMD is a late-onset inherited neuromuscular disorder, the disease generally begins in fifth decade of life or even older, and the percentage of the PABPN1 aggregates in muscle cell nuclei increases with age (63). However, due to the limited and short cycle of cultured cells, PABPN1-17A cannot form insoluble and solid-like aggregates within the cell culture period. Nevertheless, research in cultured cells may provide informative data in supporting the aggregation and sequestration models for the PABPN1 proteinopathy.

We propose that the Ala-stretch length larger than the threshold (*e.g.*, 17A ∼ 24A) may trigger PABPN1 phase transition to aggregates and sequester cellular essential factors especially the components of pre-mRNA 3′-UTR processing complexes, being implicated in a unique proteinopathy of the pathogenic PABPN1-17A in OPMD, a muscle-specific disease. There is a possibility that different cell type may have different threshold of Ala repeats for PABPN1 undergoing phase transition and thereby progressing proteinopathy. Our results may provide a line of evidence to address such a question why the disease happens only in the muscles. Thus, aggregation of Ala-expanded PABPN1 and sequestration of cellular essential factors may be fundamental to understanding of the impairment of pre-mRNA 3′-UTR processing and its relevant disease progression.

## Experimental procedures

### Plasmids, antibodies, and oligonucleotides

The DNA sequences encoding PABPN1 and several species with different length of Ala residues at the N-terminus (10A, 3A, 17A, 24A, 31A) were cloned into a FLAG-pcDNA3.1 vector *via* BamH I/Xho I sites. For PABPN1-ΔCT, the cDNAs encoding the sequences of PABPN1 (residues 1–254) were PCR amplified from the FLAG-PABPN1 plasmids using the primers of FLAG-BamH-F and ΔCT-Xho-R, then the DNA fragments were inserted into a FLAG-pcDNA3.1 vector respectively *via* BamH I/Xho I. The RRM mutants were generated by site-directed mutagenesis in the FLAG-PABPN1 plasmids using the primers of F215A/Y217A-F and F215A/Y217A-R. The EGFP-PABPN1 plasmids were generated by replacing the FLAG tag in FLAG-pcDNA3.1 vector with the EGFP sequence *via* Hind Ⅲ/BamH I. For prokaryotic expression, the cDNAs encoding PABPN1-ΔCT sequences were PCR amplified from the EGFP-PABPN1 plasmids using the primers of Nde-GFP-F and ΔCT-Xho-R, then the DNA fragments were inserted into a pET-22b vector respectively *via* Nde I/Xho I. The cDNA encoding CFIm25 was cloned into an HA-pcDNA3.1 vector *via* BamH I/Xho I. For *PAK1d*-*MLL* luciferase reporter, the distal PAS region of *PAK1* 3′-UTR was PCR-amplified from a cDNA library using the primers of PAK1d-F and PAK1d-R, and a minimized *MLL* 3′-UTR was PCR amplified from the cDNA library using the primers of MLL-F and Kpn-MLL-UTR-R. The *PAK1d*-*MLL* DNA fragment was generated by Overlap PCR using the primers of PAK1d-F and Kpn-MLL-UTR-R and then cloned into the pmirgol1 vector *via* Xba I/Kpn I. All the constructs we have cloned were validated by DNA sequencing (Table S1). The cloning vectors were from in-house stocks, and the primers were provided by technical service (Table S2). The FISH probes, ssDNAs and siRNAs, and their nucleotide sequences applied in this study are listed in Table S3. All the primary antibodies used in this study were from suppliers (Table S4), and the secondary antibodies were from Jackson ImmunoResearch Laboratories.

### Cell culture, transfection, and Western blotting analysis

HEK 293T cells and HeLa cells (Cell Bank of Chinese Academy of Sciences) were cultured in Dulbecco’s modified Eagle’s medium (HyClone) supplemented with 10% fetal bovine serum (Gibco) and penicillin-streptomycin at 37 °C under a humidified atmosphere containing 5% CO_2_. Transfection of plasmids was performed by using PolyJet reagent (SignaGen) following the manufacturer’s instructions, while transfection of ssDNA or siRNA was carried out by using Lipofectamine 3000 (Invitrogen). Protein samples or fractions from cell lysates were subjected to SDS-PAGE and then transferred onto PVDF membranes (Millipore). When needed, the blots were cut prior to antibody incubation. The indicated proteins were detected with specific primary and secondary antibodies and an ECL detection kit (Thermo Fisher Scientific). For quantification, the integral grayscale values of protein bands in gels were recorded by using *Sage Capture* software (http://www.sagecreation.com.cn/en/).

### S/P fractionation

S/P fractionation was performed as previously described (39). About 48 h after transfection, the HEK 293T or HeLa cells were harvested and lysed in 100 μl of a RIPA buffer (50 mM Tris–HCl, pH 7.5, 150 mM NaCl, 1 mM EDTA, 1% NP-40, and cocktail protease inhibitor (Roche)) on ice for 30 min, and then the lysates were centrifuged at 13,000 rpm for 15 min at 4 °C. The 90-μl supernatant was added with 30 μl of 4× loading buffer (8% SDS), while the pellet was sufficiently washed with the RIPA buffer three times at 4 °C and then added with 60 μl of 4× loading buffer (8% SDS). Equal volumes of the supernatant and pellet fractions were subjected to SDS-PAGE and Western blotting analysis. When needed, RNase A (Invitrogen) with a final concentration of 0.5 μg/μl or RNase R (Beyotime Biotechnology) with a final unit of 0.5 U/μl was included in the lysis buffer for digesting RNA.

### Immunoprecipitation and ssDNA treatment

Immunoprecipitation was performed as described (39). About 48 h after transfection, the HEK 293T cells were harvested and lysed in a PEB buffer (10 mM Hepes, pH 7.4, 100 mM KCl, 5 mM MgCl_2_, 1 mM DTT, 0.1% NP-40, 1 mM PMSF, supplemented with cocktail protease inhibitor (Roche)) on ice for 30 min, and then the lysates were centrifuged at 13,000 rpm for 20 min at 4 °C. The supernatant was added into the anti-FLAG beads (Abmart) that had been previously washed and then incubated for 4 h at 4 °C. The beads were then washed with the PEB buffer three times and boiled in 50 μl of 2× loading buffer (4% SDS). Then, the proteins were analyzed by immunoblotting. Similar experiments were also performed under the conditions of RNase A and/or ssDNA treatments. Two pieces of chimeric ssDNA (Table S4) were used in this study with a final concentration of ∼2.5 μM.

### Protein purification

The expression plasmid for each EGFP-PABPN1-ΔCT-His species was transformed into BL21 (DE3) competent *Escherichia coli* cells. The cells were grown at 37 °C till an A_600_ of 0.6 ∼ 0.8, then IPTG (0.2 mM) was added for inducing protein expression. After culture overnight at 22 °C, the cells were harvested and lysed with a lysis buffer (50 mM NaH_2_PO_4_, 500 mM NaCl, 1 mM PMSF, pH 8.0) by sonication. The cell lysates were added with RNase A and stayed on ice for 30 min, followed by centrifugation at 12,000 rpm for 30 min at 4 °C. Then the supernatant was loading onto an Ni^2+^-affinity column (Roche) for purification. After washing with the lysis buffer three times, proteins were eluted by using an elution buffer (50 mM NaH_2_PO_4_, 500 mM NaCl, 250 mM imidazole, pH 7.4). The proteins were concentrated using a 30-K centrifugal filter device (Millipore) and stored in −80 °C for further use.

### Electrophoretic mobility shift assay

For EMSA, the concentrated proteins were diluted and buffer changed with a buffer (50 mM Tris–HCl, 150 mM NaCl, 1 mM DTT) and then centrifuged at 12,000 rpm for 30 min to remove any aggregates. The protein concentration was measured by BCA Protein Assay kit (Sangon) following the manufacturer’s instructions. Two nanomolars of Cy5-labeled dA50 (Table S4) was mixed with different concentrations of the protein in a reaction buffer (50 mM Tris–HCl, 150 mM NaCl, 2 mM MgCl_2,_ pH 7.5) and incubated for 30 min at room temperature. The reaction mixtures were mixed with loading dye and subjected to electrophoresis on a 6% DNA PAGE gel. The gels were imaged using fluorescence mode on a Typhoon scanner (GE Healthcare).

### *In-vitro* PS assay

For PS assays *in vitro*, the imaging chambers were prepared following the protocol described previously (64). Briefly, the coverslip was attached to the glass slide using paralleled double-sided tapes, and then small amounts of the protein samples were put in the gap between coverslip and glass slide. Before droplet formation, the concentrated proteins were experienced buffer exchange by centrifugal filtering and concentration determination using BCA Protein Assay kit. The proteins were diluted to a final concentration of 5 μM in a PS buffer (50 mM Tris–HCl, 120 mM NaCl, 2 mM MgCl_2_, 10% PEG 8000, pH 7.5) and incubated for 30 min at room temperature, followed by centrifugation at 12,000 rpm for 3 min to remove any aggregates. For dA50-induced PS, the protein samples (5 μM) were incubated with an indicated concentration of dA50 and/or 10% Cy5-labeled dA50 in the PS buffer and incubated for 30 min at room temperature. Then, the protein samples were put onto imaging chambers and visualized by confocal microscopy (Leica TCS SP8 WLL).

### Immunofluorescence imaging and fluorescence *in situ* hybridization

For immunofluorescence imaging, about 48 h or indicated culture time after transfection, HeLa cells grown on glass coverslips were washed with a PBS buffer (10 mM Na_2_HPO_4_, 1.8 mM KH_2_PO_4_, 140 mM NaCl, 2.7 mM KCl, pH 7.3) and fixed with 4% paraformaldehyde for 15 min. After washing with the PBS buffer three times, the cells were permeabilized with 0.1% Triton X-100 and blocked with the blocking solution (5% bovine serum albumin in PBS buffer) for 1 h at room temperature. Then, the fixed cells were incubated with the respective primary antibodies overnight at 4 °C. After washing with the PBS buffer, the cells were incubated with an fluorescein isothiocyanate-conjugated antibody or a tetramethylrhodamine isothiocyanate-conjugated antibody (Jackson ImmunoResearch Laboratories). The nuclei were stained with Hoechst 33342 (Thermo Fisher Scientific). The cells were visualized on a Leica TCS SP8 WLL confocal microscope (Leica Microsystems).

For FISH, 48 h after transfection, HeLa cells grown on glass coverslips were washed with the PBS buffer and fixed with 4% paraformaldehyde for 15 min at room temperature. After washing with the PBS buffer three times, the cells were permeabilized with 0.1% Triton in 1xPBS for 15 min and washed with the PBS buffer three times again, then washed with 2× SSC (Sangon) for 10 min at room temperature. The fixed cells were incubated with the Cy5-dT25 probe (Table S4) with a final concentration of 5 pg/μl in a hybridization buffer (10% dextran sulfate sodium salt (Sigma), 10% 20× SSC (Sangon), 20% formamide (Sigma), 2 mg/ml bovine serum albumin, 1 mg/ml yeast tRNA (Sigma)) overnight at 37 °C. After successively washing with 2× SSC (Sangon) twice (each for 15 min) and 0.5× SSC and PBS (each for 15 min) at room temperature, the fixed cells were subjected to immunofluorescence imaging as the procedures described above.

### Fluorescence recovery after photobleaching

For FRAP experiment, HeLa cells were plated on a 29-mm dish with 20-mm glass-bottom well (Cellvis). About 36 h after transfection with EGFP-PABPN1 and its variants, the growth medium was changed. The cells were then imaged on an inverted laser scanning confocal microscope (Leica TCS SP8 WLL). Random choose of a circular spot with about 1.5-μm^2^ area as the bleach spot and five frames were acquired before bleaching with 2-s intervals as baseline fluorescence of the bleach spot using the 488-nm laser with 10% intensity. Then the spot was bleached for five times by 488-nm laser with 80% intensity. The recovery was monitored every 3 s for 50 frames after bleaching. The fluorescence intensities of the bleach spots were recorded by Leica microsystem (LAS X).

For *in-vitro* FRAP assay of droplets, proteins were diluted to a final concentration of 20 μM in the PS buffer and incubated for 30 min at room temperature, followed by centrifugation at 12,000 rpm for 3 min to remove any aggregates; the protein samples were put on the imaging chambers that had been previously prepared. Imaging of the chambers was done as described above. Random choose of a droplet with a circular spot of about 1-μm^2^ area as the bleach spot and five frames were acquired before bleach with 2-s intervals as baseline fluorescence of the bleach spot using the 488-nm laser with 0.3% intensity. Then the bleach spot was bleached for two times by 488-nm laser with 30% intensity. The recovery was monitored every 3 s for 50 frames after bleaching.

### Luciferase reporter assay

The *PAK1d*-*MLL* plasmid for luciferase reporter assay was constructed following the literature (4). Briefly, HEK 293T cells were transfected with the indicated plasmids or siRNAs and then 24 h later, transfected with the *PAK1d*-*MLL* plasmid. For the rescue assay, the indicated plasmids were first cotransfected into HEK 293T cells, and then 24 h later, the reporter plasmid was transfected. After culture for another 24 h, the luciferase activity was measured by using a Dual-Luciferase Reporter Assay (YEASEN) according to manufacturer’s introduction. Simultaneously, the supernatant fractions of cell lysates were subjected to SDS-PAGE and Western blotting analysis.

### Quantification of 3′-UTR APA by RNase H alternative polyadenylation assay

The method for quantifying 3′-UTR APA by RNase H alternative polyadenylation assay was as described (48). Briefly, the PABPN1-10A or PABPN1-24A plasmid was transfected into HEK 293T cells. After 48 h, the cells were harvested and the total RNA was isolated by TRIzol (Ambion) reagent. Then, 2 μg of total RNA and 50 μM gene-specific antisense DNA oligonucleotides (Table S3) were annealed before RNase-H digestion. The oligo dT primers were used to convert poly(A)-contained transcripts to cDNA. The usages of both dPAS and pPAS were quantified by RT-PCR assay with specific primer pairs. The PCR products were analyzed by 1.5% agarose gel electrophoresis.

### Quantification and statistical analysis

For quantification of Western blots, the integral grayscale values of indicated protein bands were obtained using ImageJ software (https://imagej.net/) and normalized to that of the respective control. For quantification of droplets, the confocal images were imported into ImageJ, and the number and diameter of droplets were recorded using its particle analysis module. The data were obtained from at least three independent experiments and then presented as mean ± SD. Statistical analysis were performed in GraphPad Prism 7.0 (https://www.graphpad.com/) using Student-t test. Differences were considered statistically significant at *p* <0.05. In all experiments, the *p*-values were labeled in the graphs as ∗ (*p* < 0.05), ∗∗ (*p* < 0.01), ∗∗∗ (*p* < 0.001) or N.S. (no significant).

Confocal images were acquired using LAS X software (https://www.leica-microsystems.com/) (Leica microsystem), and all images were displayed without any modification. For colocalization analyses of indicated proteins, the fluorescence intensities of respective channels were recorded using ImageJ software and plotted by GraphPad Prism 7.0. For FRAP, the fluorescence intensities of the bleach spots were recorded by LAS X (Leica microsystem). The fluorescence intensity of individual bleach spot was normalized to its mean of prebleach, and the data were plotted by using a software of OriginPro 2018 (https://www.originlab.com/) or GraphPad Prism 7.0. The mobility fraction of individual bleach spot was calculated as the following formula: Fm% = (F_1_−F_0_)/(F_∞_−F_0_) × 100%. Here, F_1_ corresponds to the mean of prebleach fluorescence intensities of the bleach spot, which is the mean fluorescence intensity of the initial five frames before bleaching; F_0_ is the fluorescence intensity of the bleach spot at bleaching; and F_∞_ is the mean fluorescence intensity of the bleach spot after recovery, which is the mean fluorescence intensity of the last five frames after recovery.

## Data availability

The data supporting the findings of this study are available from the corresponding author upon reasonable request. Source data for the figures and supplementary figures are provided as a Source Data file.

## Supporting information

This article contains supporting information.

## Conflict of interest

The authors declare that they have no conflicts of interest with the contents of this article.
