# Supplementary material for: PABPN1 aggregation is driven by Ala expansion and poly(A)-RNA binding, leading to CFIm25 sequestration that impairs alternative polyadenylation
Source: J Biol Chem. 2023 Jul 7;299(8):105019. doi: 10.1016/j.jbc.2023.105019 (PMC10403730; doi:10.1016/j.jbc.2023.105019)
Supplement: Supporting Figures S1–S5 and Tables S1–S4 [file mmc1.pdf]

PABPN1 aggregation is driven by Ala expansion and poly(A)-RNA binding, leading to CFIm25 sequestration that impairs alternative polyadenylation

Wen-Liang Guan[1,2], Lei-Lei Jiang[1], Xiao-Fang Yin[1,2], Hong-Yu Hu[1,*]

1 State Key Laboratory of Molecular Biology, Shanghai Institute of Biochemistry and Cell Biology, Center for Excellence in Molecular Cell Science, Chinese Academy of Sciences, Shanghai 200031, P. R. China.

2 University of Chinese Academy of Sciences, Beijing 100049, P. R. China.

* For correspondence: Hong-Yu Hu, hyhu@sibcb.ac.cn.

Running Title：Phase transition of PABPN1

Figure S1

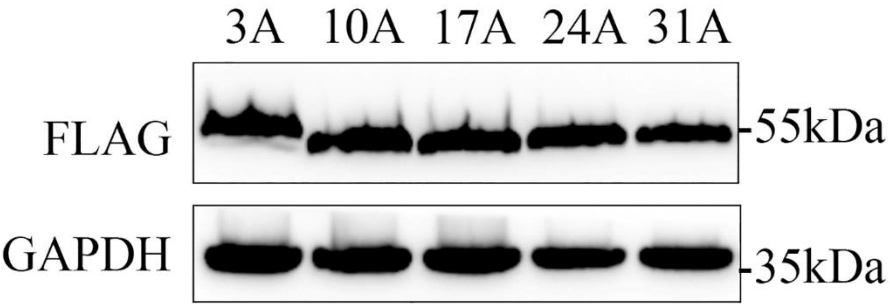

Figure S1. The total protein levels of various PABPN1 variants. FLAG-tagged PABPN1 variants (3A, 10A, 17A, 24A, 31A) were overexpressed in HeLa cells, and after 48-hr culture, the cells were lysed with RIPA buffer supplemented with 8M urea. The cell lysates were added with 4×loading buffer and subjected to SDS-PAGE and Western blotting.

Figure S2

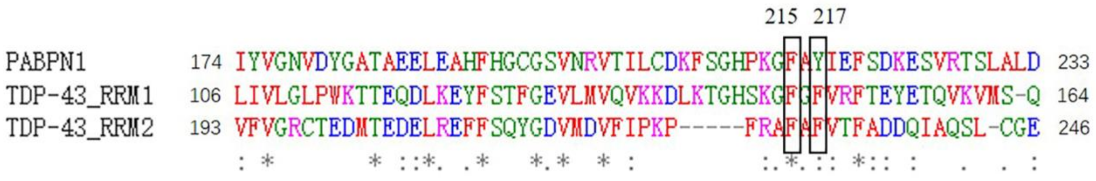

Figure S2. Sequence alignment of the RRM domains from PABPN1 and TDP-43. The sequences of the RRM domain (residues 174-233) from human PABPN1 (ID: AAH10939) and the RRM1 (106-164) and RRM2 (193-246) domains from human TDP-43 (ID: NP_031401) were aligned by the EMBL-EBI Clustal Omega website. The conserved aromatic residues that are thought to be responsible for RNA binding were highlighted by boxes. Thus, a double-point mutant (F215A/Y217A) of PABPN1 was generated to attenuate its RNA binding ability.

Figure S3

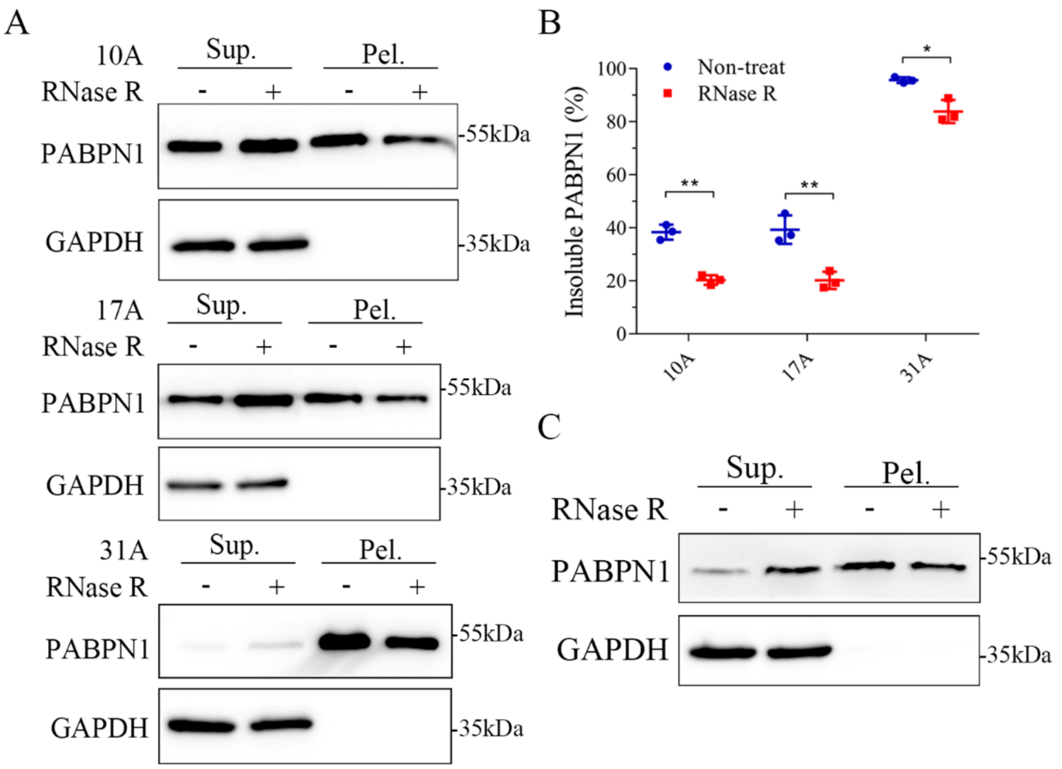

Figure S3. Effect of RNase-R treatment on the protein partitioning of PABPN1 variants. (A) S/P fractionation experiment under the condition of RNase R. FLAG-tagged PABPN1 variants (10A, 17A, 31A) were overexpressed in HeLa cells, and after 48-hr culture, the cell lysates with or without RNase-R treatment were subjected to S/P fractionation and Western blotting. (B) Quantitative analysis of the insoluble fractions in (A). F_P (%) = P/(2S+P)*100%. Data are shown as Mean ± SD (n=3). *, p<0.05, **, p<0.01. (C) S/P fractionation analysis of endogenous PABPN1 with or without RNase-R treatment.

Figure S4

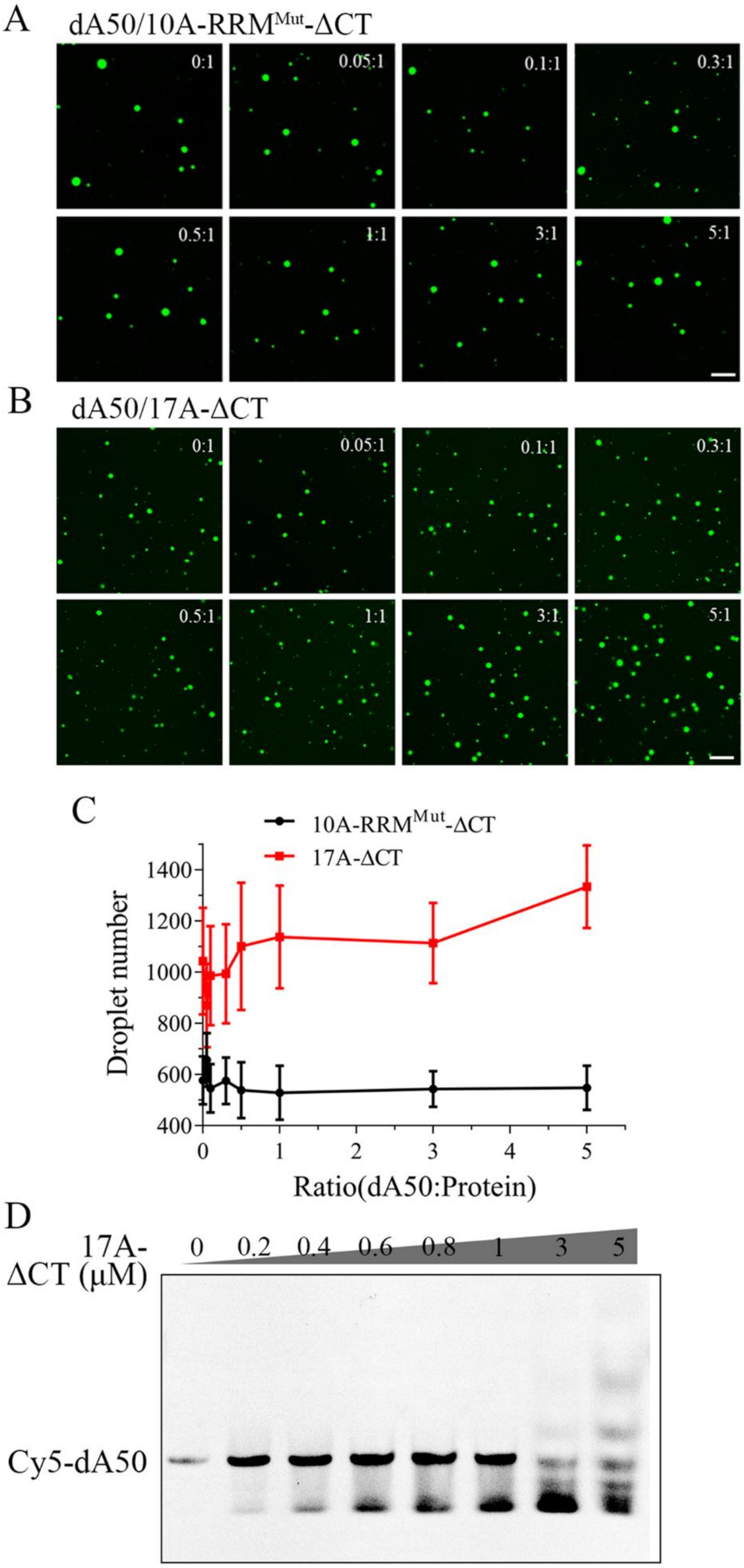

A
dA50/10A-RRM^Mut-ΔCT
0:1
0.05:1
0.1:1
0.3:1
0.5:1
1:1
3:1
5:1
B
dA50/17A-ΔCT
0:1
0.05:1
0.1:1
0.3:1
0.5:1
1:1
3:1
5:1
C
10A-RRM^Mut-ΔCT
17A-ΔCT
Droplet number
1400
1200
1000
800
600
400
0
1
2
3
4
5
Ratio(dA50:Protein)
D
17A-ΔCT (μM)
0
0.2
0.4
0.6
0.8
1
3
5
Cy5-dA50

Figure S4. Poly(A) nucleotide modulates droplet condensation of PABPN1 *in vitro*. (A, B) *In-vitro* droplet formation of the PABPN1 variants with various concentrations of dA50. Each 5 μM of EGFP-tagged PABPN1-10A-RRM^{Mut}-ΔCT (A) or PABPN1-17A-ΔCT (B) was incubated with indicated concentration of dA50 in a solution (120 mM NaCl and 10% PEG) for 30 min and imaged with confocal microscopy. Scale bar, 10 μm. (C) Quantification of the droplet numbers in (A) and (B). Data are shown as Mean ± SD (n=5). (D) EMSA for dA50 binding to PABPN1-17A-ΔCT. Cy5-labeled dA50 (2 nM) was incubated with the increasing amount of PABPN1-17A-ΔCT.

Figure S5

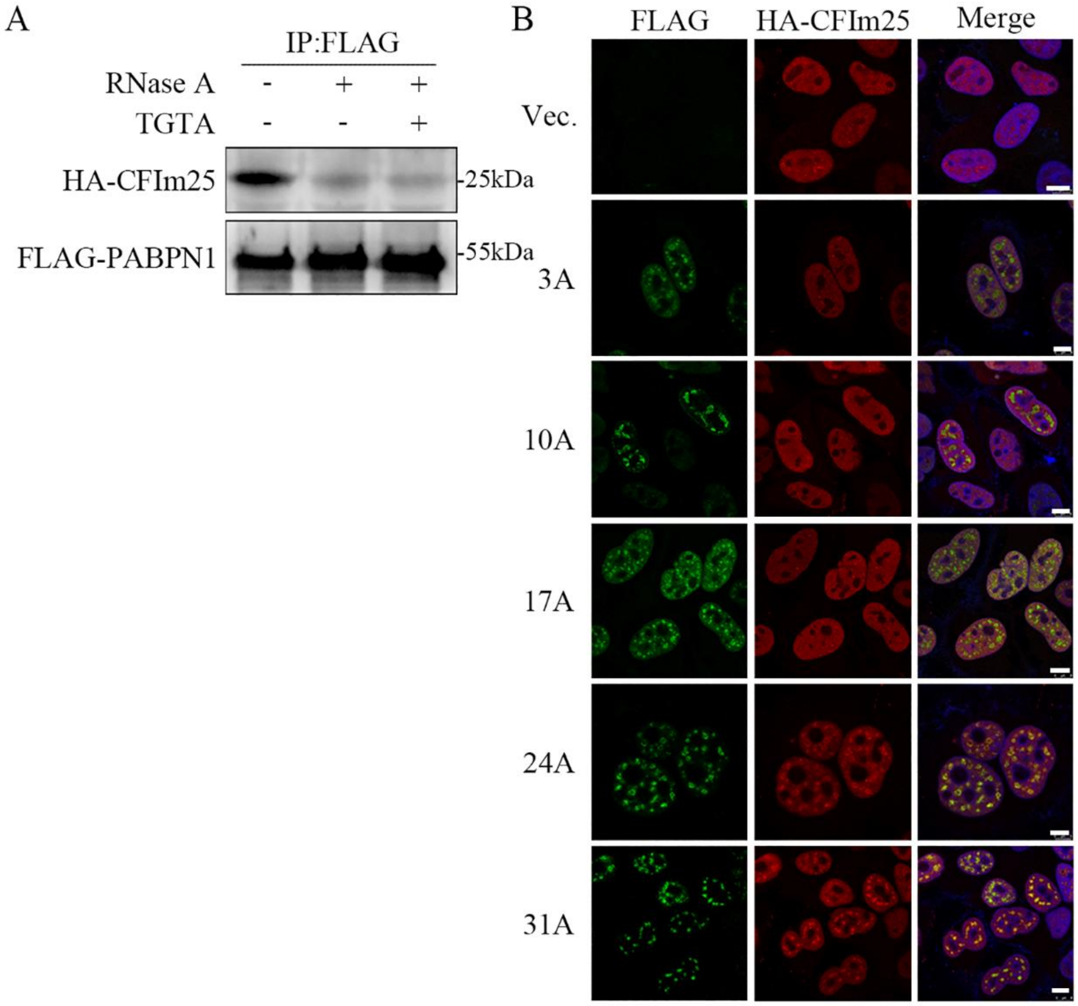

Figure S5. The PABPN1 aggregates sequester CFIm25. (A) Co-IP analysis under the conditions of RNase A and TGTA ssDNA treatments for interaction between PABPN1 and CFIm25. RNase A and/or TGTA were added into the cell lysates before immunoprecipitation. (B) Immunofluorescence imaging of the PABPN1 variants with exogenous CFIm25. HeLa cells were co-transfected with FLAG-tagged PABPN1 and HA-CFIm25, after 48-hr culture, the cells were fixed and immunostained with anti-FLAG (green) and anti-HA (red) antibodies. Nuclei were stained with Hoechst (blue). Scale bar, 10 μm.

Table S1. List of the constructs applied in this study.

Constructs	Vectors	Restriction Enzyme sites	Additional
FLAG-PABPN1-10A	FLAG-pcDNA3.1	BamH I / Xho I	10 Ala, FLAG tag
FLAG-PABPN1-3A	FLAG-pcDNA3.1	BamH I / Xho I	3 Ala
FLAG-PABPN1-17A	FLAG-pcDNA3.1	BamH I / Xho I	17 Ala
FLAG-PABPN1-24A	FLAG-pcDNA3.1	BamH I / Xho I	24 Ala
FLAG-PABPN1-31A	FLAG-pcDNA3.1	BamH I / Xho I	31 Ala
EGFP-PABPN1-10A	FLAG-pcDNA3.1	Hind III / Xho I	EGFP fusion
EGFP-PABPN1-3A	FLAG-pcDNA3.1	Hind III / Xho I	EGFP fusion
EGFP-PABPN1-17A	FLAG-pcDNA3.1	Hind III / Xho I	EGFP fusion
EGFP-PABPN1-24A	FLAG-pcDNA3.1	Hind III / Xho I	EGFP fusion
EGFP-PABPN1-24A	FLAG-pcDNA3.1	Hind III / Xho I	EGFP fusion
FLAG-PABPN1-10A-RRM^{Mut}	FLAG-pcDNA3.1	BamH I / Xho I	F215A/Y217A
FLAG-PABPN1-3A-RRM^{Mut}	FLAG-pcDNA3.1	BamH I / Xho I	F215A/Y217A
FLAG-PABPN1-17A-RRM^{Mut}	FLAG-pcDNA3.1	BamH I / Xho I	F215A/Y217A
FLAG-PABPN1-24A-RRM^{Mut}	FLAG-pcDNA3.1	BamH I / Xho I	F215A/Y217A
FLAG-PABPN1-31A-RRM^{Mut}	FLAG-pcDNA3.1	BamH I / Xho I	F215A/Y217A
FLAG-PABPN1-10A-ΔCT	FLAG-pcDNA3.1	BamH I / Xho I	Residues 1-254, C-terminal deletion
FLAG-PABPN1-10A-RRM^{Mut}-ΔCT	FLAG-pcDNA3.1	BamH I / Xho I	Residues 1-254, F215A/Y217A
FLAG-PABPN1-17A-ΔCT	FLAG-pcDNA3.1	BamH I / Xho I	Residues 1-254
FLAG-PABPN1-31A-ΔCT	FLAG-pcDNA3.1	BamH I / Xho I	Residues 1-254
EGFP-PABPN1-10A-ΔCT-His	pET-22b	Nde I / Xho I	Prokaryotic
EGFP-PABPN1-10A-RRM^{Mut}-ΔCT-His	pET-22b	Nde I / Xho I	Prokaryotic F215A/Y217A
EGFP-PABPN1-17A-ΔCT-His	pET-22b	Nde I / Xho I	Prokaryotic
PAPα-Myc	pcDNA3.1-Myc	BamH I / Xho I	C-terminal Myc tag
HA-CFIm25	HA-pcDNA3.1	BamH I / Xho I	N-terminal HA tag
PAK1d-MLL	pmirgol1	Xba I /Kpn I	Luciferase reporter

Table S2. Nucleotide sequences of PCR primers applied in this study.

PCR primer	Sequence	Note
FLAG-BamH-F	gactacaaagacgatgacga	BamH I site
ΔCT-Xho-R	ccgctcgagttagatgcctggtctgttggttc	Xho I site
F215A/Y217A-F	ggccatcccaaaggggctgcggctatagagttctcagac	Mutagenesis
F215A/Y217A-R	gtctgagaactctatagccgcagcccctttgggatggcc	Mutagenesis
Nde-GFP-F	ggaattccatatggtgagcaagggcgag	Nde I site
ΔCT-Xho-R	ccgctcgaggatgcctggtctgttggt	Xho I site,<br>No stop codon
BamH-PAP-F	cgcggatccatgccgtttccagttaca	BamH I site
Xho-PAP-R	ccgctcgagccgattcaatctcagttt	Xho I site,<br>No stop codon
BamH-CFIm25-F	cgcggatccatgtctgtggtaccgccc	BamH I site
Xho-CFIm25-R	ccgctcgagtcagttgtaaataaaattgaacctgctca	Xho I site
PAK1d-F	tagcctcgagtctagaaatgcttacactgggtgt	
PAK1d-R	cccactttctaacaggaatattttcatctcctt	
MLL-F	ctgttagaaagtgggaat	
Kpn-MLL-UTR-R	cggggtacctagtgttctgccttcaac	Kpn I site,<br>MLL 3′-UTR
Pak1 distal-F	gaaatgcttacactgggtgt	For RT-PCR
Pak1 distal-R	tgtgctgcagaggcagtagt	For RT-PCR
Pak1 proximal-F	tgtgtgccgtgagtgtctg	For RT-PCR
Pak1 proximal-R	gccatccagtatggggttcc	For RT-PCR

Table S3. Nucleotide sequences of FISH probes, ssDNAs and siRNAs applied in this study.

Nucleotide	Sequence	Note
Probe		Cy5 labeled
Cy5-dT25	Cy5-5′-TTTTTTTTTTTTTTTTTTTTTTTTT-3′	oligo-dT
Cy5-dA50	Cy5-5′-<br>AAAAAAAAAAAAAAAAAAAAAAAAAAAAAAAA<br>AAAAAAAAAAAAAAAAAA-3′	poly(dA)
ssDNA		
dA50	AAAAAAAAAAAAAAAAAAAAAAAAAAAAAAAA<br>AAAAAAAAAAAAAAAAAA	poly(dA)
TGTA	GGGTGTAAACAGATGATGTATAAAAAAAAAAAAA<br>AAAAAAAAAAAAAAAAAAAAAAAAA	TGTA repeat
dUGdUA	GGG<u>dU</u>G<u>dU</u>AAACAGA<u>dU</u>GA<u>dU</u>G<u>dU</u>A<u>dU</u>AAAAAAA<br>AAAAAAAAAAAAAAAAAAAAAAAAAAAAAAA	dUGdUA repeat
ExF2-RNaseH	CCCGACTTCTCAACACTGCT	For RHAPA
siRNA		
siRNA-CFIm25	5′-ACCUCCUCAGUAUCCAUAUtt-3′<br>5′-AUAUGGAUACUGAGGAGGUUC-3′	

Table S4. List of the antibodies used in this study.

Antibody	Source	Catalog No.
Anti-FLAG (mouse)	Sigma-Aldrich	Cat# F1804
Anti-HA (mouse)	Sigma-Aldrich	Cat# H9658
Anti-Myc (mouse)	Cell Signaling Technology	Cat# 2276S
Anti-PABPN1 (rabbit)	Abcam	Cat# ab75855
Anti-CFIm25 (mouse)	Santa Cruz Biotechnology	Cat# sc-81109
Anti-SC35 (mouse)	Sigma-Aldrich	Cat# S4045
Anti-GAPDH (mouse)	Proteintech	Cat# 60004-1-1g
Peroxidase AffiniPure Goat Anti-Mouse IgG (H+L)	Jackson ImmunoResearch Laboratories	Cat# 115-035-003
Peroxidase AffiniPure Goat Anti-Rabbit IgG (H+L)	Jackson ImmunoResearch Laboratories	Cat# 111-001-003
FITC IgG Fluorescein-Conjugated AffiniPure Goat Anti-Rabbit IgG(H+L)	ZSGB-BIO	Cat# ZF-0311
TRITC IgG Fluorescein-Conjugated AffiniPure Goat Anti-Rabbit IgG(H+L)	ZSGB-BIO	Cat# ZF-0316
FITC IgG Fluorescein-Conjugated AffiniPure Goat Anti-Mouse IgG(H+L)	ZSGB-BIO	Cat# ZF-0312
TRITC IgG Fluorescein-Conjugated AffiniPure Goat Anti-Mouse IgG(H+L)	ZSGB-BIO	Cat# ZF-0313
